# Genomic Therapy Matching in Rare and Refractory Cancers

**DOI:** 10.1001/jamaoncol.2026.0127

**Published:** 2026-03-05

**Authors:** Frank P. Lin, Subotheni Thavaneswaran, John P. Grady, Christine E. Napier, Maya Kansara, Lucille Sebastian, Damien Kee, Jayesh Desai, Milita Zaheed, Sarah Chinchen, Samantha R. Oakes, James Blackburn, Hamish S. Scott, Anthony Glover, Stephen B. Fox, David Goldstein, Paul Leo, Benhur Amanuel, Antony Mersiades, Michael Millward, Michael P. Brown, Michail Charakidis, Adrian M. J. Pokorny, Paul Craft, David Espinoza, Peter Grimison, Rosemary Harrup, Anthony M. Joshua, Ken O’Byrne, Chee Khoon Lee, Mark J. Cowley, Mandy L. Ballinger, John Simes, David M. Thomas, and the Australian Molecular Screening and Therapeutic program investigators and contributors

**Affiliations:** 1 NHMRC Clinical Trials Centre, University of Sydney, Camperdown, New South Wales, Australia; 2 Garvan Institute of Medical Research, Sydney, New South Wales, Australia; 3 School of Clinical Medicine, UNSW Sydney, Kensington, New South Wales, Australia; 4 The Kinghorn Cancer Centre, St Vincent’s Hospital Sydney, Darlinghurst, New South Wales, Australia; 5 Rare Cancer Laboratory, Walter and Eliza Hall Institute of Medical Research, Melbourne, Victoria, Australia; 6 Department of Medical Oncology, Peter MacCallum Cancer Centre, Melbourne, Victoria, Australia; 7 Department of Medical Oncology, Austin Health, Heidelberg, Victoria, Australia; 8 Prince of Wales Hereditary Cancer Clinic, Prince of Wales Hospital, Randwick, New South Wales, Australia; 9 National Breast Cancer Foundation, Sydney, New South Wales, Australia; 10 Department of Genetics and Molecular Pathology, SA Pathology, Adelaide, South Australia, Australia; 11 Centre for Cancer Biology, SA Pathology and University of South Australia, Adelaide, South Australia, Australia; 12 Faculty of Medicine and Health, University of Sydney School of Medicine, Camperdown, New South Wales, Australia; 13 Department of Pathology, Peter MacCallum Cancer Centre and University of Melbourne, Melbourne, Victoria, Australia; 14 Department of Medical Oncology, Prince of Wales Hospital, Randwick, New South Wales, Australia; 15 Centre for Genomics and Personalized Health, Queensland University of Technology, Brisbane, Queensland, Australia; 16 School of Pathology and Laboratory Medicine, University of Western Australia, Perth, Western Australia, Australia; 17 PathWest Laboratory Medicine, Queen Elizabeth II Medical Centre, Nedlands, Western Australia, Australia; 18 School of Medical Science, Edith Cowan University, Joondalup, Western Australia, Australia; 19 Department of Medical Oncology, Northern Beaches Hospital, Frenchs Forest, New South Wales, Australia; 20 School of Medicine, University of Western Australia, Perth, Western Australia, Australia; 21 Linear Clinical Research, Nedlands, Western Australia, Australia; 22 Cancer Clinical Trials Unit, Royal Adelaide Hospital, School of Medicine, University of Adelaide, Adelaide, South Australia, Australia; 23 Department of Medical Oncology, Royal Darwin Hospital, Tiwi, Northern Territory, Australia; 24 Department of Medical Oncology, Alice Springs Hospital, Gap, Northern Territory, Australia; 25 Medical Oncology Department, Canberra Region Cancer Centre, Garran, Australian Capital Territory, Australia; 26 Chris O’Brien Lifehouse, Camperdown, New South Wales, Australia; 27 Department of Medical Oncology, Royal Hobart Hospital, Hobart, Tasmania, Australia; 28 Department of Medical Oncology, Princess Alexandra Hospital, Brisbane, Queensland, Australia; 29 Queensland University of Technology, Brisbane, Queensland, Australia; 30 Department of Medical Oncology, St George Hospital, Kogarah, New South Wales, Australia; 31 Children’s Cancer Institute, Randwick, New South Wales, Australia; 32 Centre for Molecular Oncology, UNSW Sydney, Kensington, New South Wales, Australia

## Abstract

**Importance:**

The clinical utility of matching therapies to genomic biomarkers based on varying levels of evidence remains uncertain, particularly for patients with rare and refractory cancers.

**Objective:**

To assess whether a tiered, evidence-based framework for matching genomic biomarkers to therapies is associated with differential overall survival in patients with advanced solid tumors.

**Design, Setting, and Participants:**

This multicenter cohort study was conducted within the Molecular Screening and Therapeutic program, a nationwide precision oncology program in Australia. Patients aged 18 years and older with advanced, refractory solid tumors and adequate Eastern Cooperative Oncology Group Performance Status were enrolled from June 2016 to December 2021, with follow-up through July 2022. Data were analyzed from July 2022 to July 2024.

**Exposures:**

Systemic therapy following comprehensive genomic profiling. Therapies were classified as matched or unmatched using the TOPOGRAPH (Therapy-Oriented Precision Oncology Guidelines for Recommending Anticancer Pharmaceuticals) knowledge base, which stratifies biomarker-drug pairs by level of evidence (tiers 1-3A, prospective trial evidence; tiers 3B/4, investigational/repurposed).

**Main Outcome and Measures:**

The primary outcome was overall survival from date of molecular profiling results. The hypothesis was tested using a time-dependent multivariable Cox proportional hazards model, adjusted for age, Eastern Cooperative Oncology Group Performance Status, cancer type, prior therapy, and prior receipt of matched therapy.

**Results:**

Of 3383 patients (mean [SD] age 57.1 [14.3] years; 1792 [53.0%] female), 1270 (37.5%) had a clinically active (tiers 1-3A) biomarker. Among patients with a tier 1 to 3A biomarker receiving treatment, those receiving matched therapy had a longer median overall survival than those receiving unmatched therapy (21.2 months [95% CI, 17.1-26.8 months] vs 12.8 months [95% CI, 11.7-13.9 months]; adjusted hazard ratio [aHR], 0.60; 95% CI, 0.44-0.82; *P* = .001). In contrast, among patients receiving therapy matched to investigational evidence (tiers 3B/4), there was not an associated survival benefit compared with unmatched therapy (14.5 months [95% CI, 12.6-18.4 months] vs 12.8 months [95% CI, 12.0-14.7 months]; aHR, 1.04; 95% CI, 0.84-1.29; *P* = .71). Patients who received therapies repurposed from other cancer types based solely on a biomarker and lacking direct evidence (tier 3B) did not experience longer survival compared with those receiving unmatched therapy (13.6 months [95% CI, 8.0-16.8 months] vs 12.5 months [95% CI, 11.3-13.5 months]; aHR, 1.40; 95% CI, 1.00-1.96; *P* = .047).

**Conclusions and Relevance:**

In this cohort study of patients with advanced solid tumors, matching therapies to genomic biomarkers was associated with improved survival only when supported by prospective clinical trial evidence. These findings support using an evidence-based framework to prioritize genomically guided therapies.

## Key Points

**Question:**

In patients with advanced, refractory cancers, is a tiered framework for matching genomic biomarkers to therapies associated with improved survival?

**Findings:**

In this cohort study of 3383 patients with advanced solid tumors, those with biomarkers supported by prospective trial evidence who received matched therapy had a median overall survival of 21.2 months vs 12.8 months for those receiving unmatched therapy. No survival benefit was observed for therapies matched using preclinical evidence or repurposed from other cancer types.

**Meaning:**

These findings support prioritizing genomically guided therapies based on the strength of clinical evidence, as only matches supported by prospective trials were associated with a survival benefit.

## Teaser

This cohort study assesses whether a tiered, evidence-based framework for matching genomic biomarkers to therapies is associated with differential overall survival in patients with advanced solid tumors.

## Introduction

Genomic profiling has fundamentally altered the therapeutic landscape in oncology, propelling biomarker-centric drug development while leading to the regulatory approval of tumor-agnostic therapies.^1,2,3,4,5,6,7,8^ Clinically, large multigene panel testing is increasingly used to identify treatment options for patients with advanced cancers who have exhausted standard therapies. Although studies using genomic biomarker selection have reported improved disease control,^9,10,11,12,13,14,15,16^ clinical outcomes remain inconsistent across different programs.^17,18,19,20,21^ A primary contributor to this variability is the lack of a standardized definition of *actionability* of genomic alterations. Methodologies for determining actionability vary widely and include the consensus of molecular tumor boards,^16,18,22,23,24,25^ algorithmic matching scores,^26^ and pathway-based analyses.^16,22^ Heterogeneous interpretation can lead to inconsistent recommendations, complicating the evaluation of clinical utility of genomic biomarker testing.^24^

We report the outcomes from the screening component of the national Molecular Screening and Therapeutic (MoST) program in Australia.^25^ The primary objective of the MoST program is to use genomic profiling of archival tissue to identify actionable biomarkers for patients with advanced, rare, or neglected cancers. To address the challenge of inconsistent biomarker interpretation, we previously developed the Therapy-Oriented Precision Oncology Guidelines for Recommending Anticancer Pharmaceuticals (TOPOGRAPH) framework.^27^ This framework is based on a curated catalog of biomarker-linked therapies, with each therapy categorized by the strength of its clinical evidence and its stage of drug development.

The primary aim of this cohort study is to report the outcomes of the MoST screening program. A key secondary aim is to determine whether the TOPOGRAPH framework can stratify clinical outcomes for patients with cancer receiving genomically matched therapy, thereby informing the development of a robust system to guide therapy selection for this population with limited treatment options.

## Methods

### Study Type and Oversight

This cohort study was conducted within the MoST program, a national precision oncology initiative,^25^ approved by the St Vincent’s Hospital Human Research Ethics Committee. All participants provided written informed consent prior to enrollment. The study is registered with the Australian New Zealand Clinical Trials Registry (ACTRN12616000908437) and followed Strengthening the Reporting of Observational Studies in Epidemiology (STROBE) reporting guidelines.

### Study Population and Eligibility

Eligible participants were adults (≥18 years old) with a pathologically confirmed metastatic or advanced solid tumor for which standard systemic therapies had been exhausted or were not tolerated. Inclusion required an Eastern Cooperative Oncology Group (ECOG) Performance Status of 0 to 2. Key exclusion criteria were uncontrolled comorbidities or symptomatic central nervous system involvement. Patients with a history of another cancer within 2 years prior to enrollment were excluded, unless the prior cancer had been definitively treated and was progression free for 6 months.

### Study Cohort

Consecutive patients who underwent genomic profiling between June 2016 and December 2021 were screened. This study period was selected to align with the nationwide implementation of a cancer genomics program that stipulated a recruitment target of 3095 patients. Patients were excluded from the analysis for poor tissue quality, sequencing failure, or death prior to the availability of genomic profiling results. Clinical and demographic data, primary diagnosis, ECOG Performance Status, and Charlson Comorbidity Index were collected at the time of consent. The number of prior lines of therapy, defined by unique systemic treatment or combinations, was determined by 3 investigators (F.P.L., S.T., and C.E.N.).

The study population was stratified into 2 cohorts based on whether subsequent systemic therapy was administered after genomic results were returned: cohort A received no further therapy, and cohort B received 1 or more lines of therapy following genomic profiling. This stratification was carried out to facilitate a meaningful analysis of treatment outcomes, as considerable heterogeneity in survival characteristics was observed across the screened population (eMethods in Supplement 1).

### Genomic Profiling, Tiering, and Therapy Matching Strategy

Archival formalin-fixed, paraffin-embedded diagnostic material was sequenced using next-generation sequencing panels, primarily the Illumina TruSight Oncology (TST170 and TSO500) and the FoundationOne CDx panels. Genomic variants were reviewed at weekly molecular tumor board meetings (fortnightly at Western Australian sites).

The level of evidence for therapy-biomarker associations was classified using the TOPOGRAPH knowledge base (version AU 20220828).^27^ Systemic therapies matched to genomic variants were stratified into hierarchical tiers: tiers 1 to 2 (approved for use with the specific biomarker in the cancer type), tier 3 (proven clinical activity in the cancer type but not yet standard care), tier 3B (therapies with established efficacy for the same biomarker in a different cancer type but lacking direct evidence in the specific histotype), tier 4 (preclinical or early clinical evidence), and tier R2 (therapies predicted to be inactive).

For patients in cohort B, the overall tier of the therapy received after genomic profiling was determined according to a predefined hierarchy for identifying the most active therapy: (1) the therapy with the highest TOPOGRAPH tier among those received subsequent to genomic profiling; (2) for multiple therapies genomically matched to the same tier, the earliest therapy administered; or (3) classifying the therapy as unmatched if it had no corresponding genomic biomarker. The tiering process was performed algorithmically to prevent subjective bias in tier assignment.^28^

### Outcome Measures

The primary outcome was overall survival (OS), defined as the time from receipt of molecular profiling results to death from any cause. Participants underwent follow-up assessments at 6 and 12 months after sequencing, then annually for up to 5 years. For the OS analysis, data were censored at July 20, 2022. Patients of unknown survival status were censored at their last follow-up date. Secondary outcomes included the frequency and type of actionable molecular alterations, the proportion of patients who received genomically matched therapy, and the molecular profiling turnaround time (days from sample receipt to report finalization).

### Statistical Analysis

Baseline characteristics were summarized using descriptive statistics: medians with IQRs for continuous variables and frequencies for categorical variables. Differences between patient groups were assessed using the Mann-Whitney U test (continuous variables) and the χ^2^ test (categorical variables).

The primary analysis compared OS between patients who received genomically matched therapy and those who received nonmatched therapy. Matched therapies were categorized into evidence tiers 1 to 4 based on the level of evidence defined in the TOPOGRAPH database. OS was estimated using the Kaplan-Meier method; between-group difference was compared using the log-rank test. Median follow-up time was calculated with the reverse Kaplan-Meier method. Prespecified subgroup analyses of OS were conducted to compare (1) patients receiving any tier-matched therapy (tiers 1-4) vs those with an actionable alteration who received only nonmatched therapy, (2) patients receiving therapies with established clinical activity (tiers 1-3) vs those receiving investigational therapies (tiers 3B/4), and (3) patients within individual tier groups (tiers 1-2, 3, 3B, and 4).

To adjust for confounders and time-dependent biases related to the timing of therapy initiation, a multivariable Cox proportional hazards model was constructed, incorporating a time-dependent covariate for the initiation of the most active matched therapy. The following baseline prognostic factors were also adjusted: age, ECOG Performance Status, cancer type, prior lines of therapy, and prior receipt of a tier-matched therapy. The primary result of this model was the adjusted hazard ratio (aHR) for death, reported with 95% CIs.

R, version 4.0.5 (R Project for Statistical Computing), was used for descriptive, survival, and regression analyses. All statistical tests were 2-sided, and *P* < .05 was considered statistically significant. Data were analyzed from July 2022 to July 2024.

## Results

### Study Population

**Figure 1.**
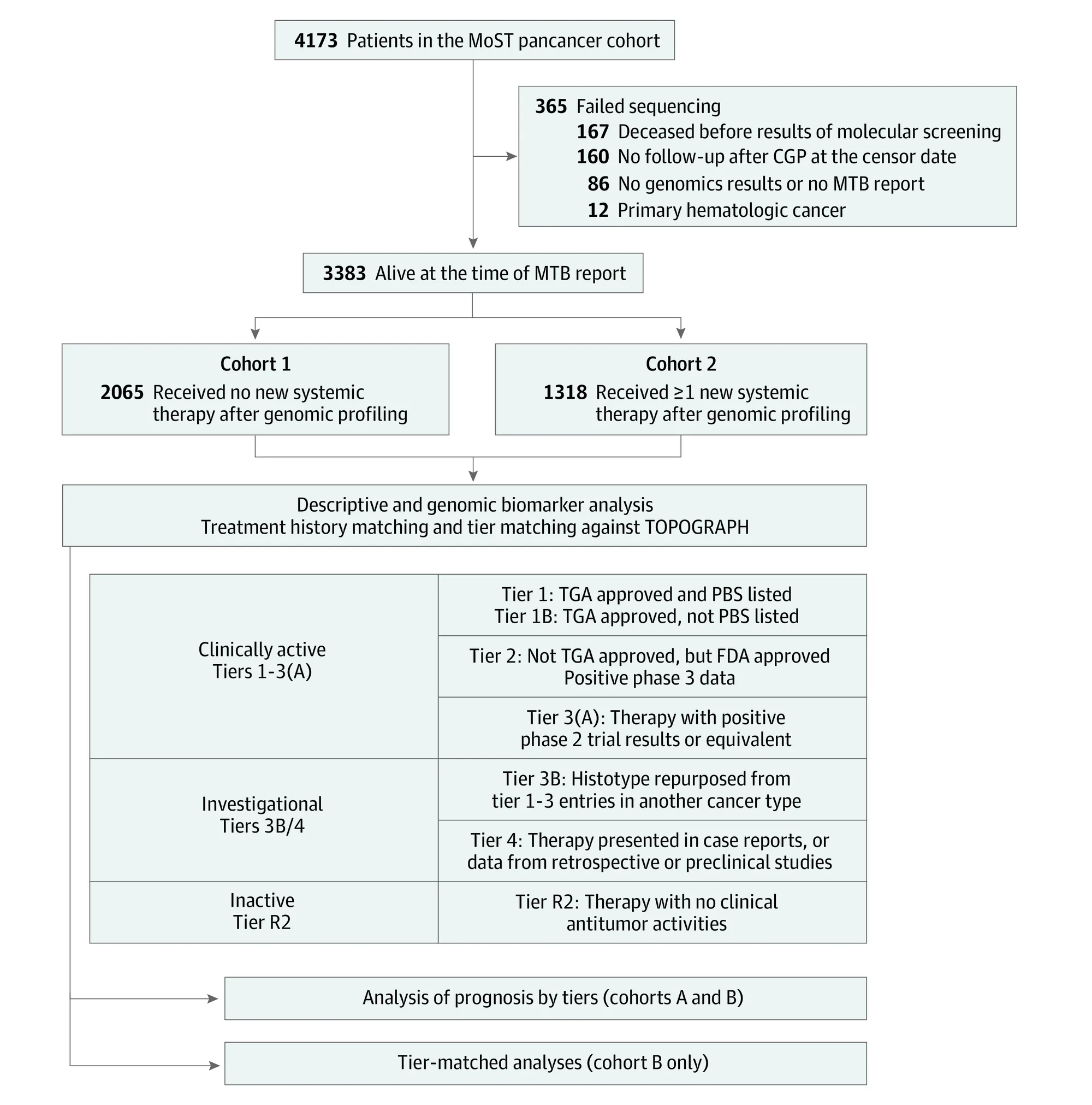
Cohort Flow Diagram and Genomic Matching Tier Definitions Prognostic evaluations were conducted for both cohorts A and B. In cohort B, tier-matched analyses were also conducted to assess the potential treatment effect of genomically matched therapies, stratified by tiers. Further exploratory analyses were conducted to identify potential genomic alterations that may be predictive of differential treatment effects. Therapies, together with their genomic profiling results and cancer type, were searched against the TOPOGRAPH (Therapy-Oriented Precision Oncology Guidelines for Recommending Anticancer Pharmaceuticals) knowledge base. This knowledge base comprises triples of biomarker variants, cancer type, and drug, all linked by supporting evidence. A therapy is considered matched if any drug from its regimen class is present in the TOPOGRAPH curation, mirroring the clinical strategy used in therapy selection based on genomic biomarker testing. CGP indicates comprehensive genomic profiling; FDA, US Food and Drug Administration; MoST, Molecular Screening and Therapeutic program; MTB, molecular tumor board; PBS, Pharmaceutical Benefits Scheme; TGA, Therapeutic Goods Administration.

The MoST program recruited 4173 heavily pretreated patients with rare and refractory advanced solid tumors between June 2016 and December 2021 (Figure 1). After exclusions, results from 3383 patients (81.1%; mean [SD] age, 57.1 [14.3] years; 1792 [53.0%] female) were eligible to be included for analysis at data cutoff. The most frequent cancers were sarcoma in 654 patients (19.3%), colorectal cancer in 346 (10.2%), pancreatic cancer in 288 (8.5%), high-grade gliomas in 239 (7.1%), and breast cancer in 189 (5.6%). Overall, 2427 patients (71.7%) had rare cancers (<6 per 100 000 population frequency), and 229 (6.8%) had less common cancers (6-12 per 100 000 population frequency) (Table and eTable 1 in Supplement 1).

**Table.** Characteristics of Cohorts With Solid Tumors in the Molecular Screening and Therapeutic Program

Characteristic	No. (%)		
	Cohort A (n = 2065)	Cohort B (n = 1318)	Total (N = 3383)
Age group, y^a^			
<20	8 (0.4)	10 (0.8)	18 (0.5)
20-34	148 (7.2)	119 (9.0)	267 (7.9)
35-44	231 (11.2)	171 (13.0)	402 (11.9)
45-54	383 (18.5)	262 (19.9)	645 (19.0)
55-64	570 (27.6)	366 (27.8)	936 (27.7)
65-74	529 (25.6)	310 (23.5)	839 (24.8)
75-84	186 (9.0)	79 (6.0)	265 (7.8)
≥85	10 (0.5)	1 (0.1)	11 (0.3)
Sex			
Female	1072 (51.9)	720 (54.6)	1792 (53.0)
Male	993 (48.1)	598 (45.4)	1591 (47.0)
ECOG group^a^			
0	884 (42.8)	706 (53.6)	1590 (47.0)
1	1072 (51.9)	575 (43.6)	1647 (48.7)
2	94 (4.6)	31 (2.4)	125 (3.7)
3	1 (<0.1)	1 (0.1)	2 (<0.1)
Not reported	1 (<0.1)	0	1 (<0.1)
Urban-rural classification^b^			
Urban	1852 (89.7)	1214 (92.1)	3066 (90.6)
Rural	212 (10.3)	102 (7.7)	314 (9.3)
Other	1 (<0.1)	2 (<0.1)	3 (<0.1)
Rare class^a^			
Common	442 (21.4)	285 (21.6)	727 (21.5)
Less common	167 (8.1)	62 (4.7)	229 (6.8)
Rare	1456 (70.5)	971 (73.7)	2427 (71.7)
Cancer type^a^			
Sarcoma	354 (17.1)	300 (22.8)	654 (19.3)
Colorectal	205 (9.9)	141 (10.7)	346 (10.2)
Pancreas	185 (9.0)	103 (7.8)	288 (8.5)
High-grade gliomas	146 (7.1)	93 (7.1)	239 (7.1)
Breast	104 (5.0)	85 (6.4)	189 (5.6)
Ovarian	90 (4.4)	82 (6.2)	172 (5.1)
Biliary and gallbladder	113 (5.5)	57 (4.3)	170 (5.1)
Gastroesophageal	105 (5.1)	44 (3.3)	149 (4.4)
Unknown primary	79 (3.8)	54 (4.1)	133 (3.9)
Non–small cell lung	78 (3.8)	36 (2.7)	114 (3.4)
Endometrial	64 (3.1)	33 (2.5)	97 (2.9)
Prostate	53 (2.6)	40 (3.0)	93 (2.7)
Bladder	44 (2.1)	32 (2.4)	76 (2.2)
Head and neck	53 (2.6)	13 (1.0)	66 (2.0)
Small bowel and appendix	42 (2.0)	20 (1.5)	62 (1.8)
Salivary gland	40 (1.9)	21 (1.6)	61 (1.8)
Kidney	31 (1.5)	15 (1.1)	46 (1.4)
Other	279 (13.5)	149 (11.3)	428 (12.7)

Abbreviation: ECOG, Eastern Cooperative Oncology Group.

^a^ χ^2^ Test for independence was used, suggesting that statistically significant difference was observed between cohorts A (patients who received no further systemic therapy) and B (patients who received at ≥1 line of further systemic therapy) at a type I error rate of .05. The full table is shown in eTable 1 in Supplement 1.

^b^ The urban and rural classification is defined by the Accessibility/Remoteness Index of Australia system, an official indicator code of remoteness used by the Australian Bureau of Statistics.

### Participant Characteristics

Eligible patients were stratified into 2 cohorts (Figure 1): 2065 patients (61.0%) who received no further systemic therapies (cohort A) and 1318 patients (39.0%) who received systemic therapy after genomic profiling (cohort B). Compared to cohort B, patients in cohort A were older (mean [SD] age, 58.0 [14.2] years in cohort A vs 55.7 [14.3] years in cohort B; *P* < .001), had worse performance status (ECOG group 0, 884 of 2065 [42.8%] in cohort A vs 706 of 1318 [53.6%] in cohort B; *P* < .001), and had greater comorbidities (mean [SD] Charlson Comorbidity Index, 1.63 [1.67] in cohort A vs 1.38 [1.52] in cohort B; *P* < .001) at enrollment. There was not a statistically significant difference in the mean (SD) number of lines of systemic therapy received prior to genomic profiling between cohorts A and B (1.71 [1.52] vs 1.70 [1.49]; *P* = .73).

The patients were followed for a median (IQR) of 22.6 (11.1–35.4) months, with 2131 deaths (63.0%). The median OS after genomic profiling was 11.3 months (95% CI, 10.5-11.9 months). A shorter median OS was observed in cohort A (8.2 months; 95% CI, 7.4-9.0 months) compared to cohort B (14.1 months; 95% CI, 13.4-15.2 months). In cohort B, the median time to first systemic therapy was 2.4 months (95% CI, 2.2-2.6 months). The median (IQR) time from study consent to the return of genomic profiling results was 49 (36-64) days (mean, 71.5 days). The median OS from study consent was 13.3 months (95% CI, 12.7-14.1 months): 10.4 months (95% CI, 9.6-11.5 months) in cohort A and 16.8 months (95% CI, 15.6-17.8 months) in cohort B. The landscape of alterations among the entire cohort is shown in eFigure 1 in Supplement 1.

### Therapy-Associated TOPOGRAPH Tiers Based on Genomic Results

There were 3003 patients (88.8%) with 1 or more genomic biomarkers for which a tier (1-4) matched therapy could be assigned (tier-assigned group). Clinically active biomarker-linked therapies (tier 1-3A) were identified in 1270 patients (37.5%). The proportion of patients with no tiered genomic biomarker (untiered group) was higher in cohort A than in cohort B (257 [12.5%] vs 123 [9.3%]; *P* = .006). In the tier-assigned group, no difference was noted in the distribution of tiers between cohorts A and B (eTable 2 in Supplement 1). For the clinically active tier group in cohort B (tiers 1-3A; 526 of 3383 patients [15.5%]), the median (IQR) of matched biomarker-linked drug classes per patient was 0 (0-1) (mean, 1.0) but with a maximum of 22 actionable drug classes. The median (IQR) number of actionable drug classes matched to the investigational therapy tier group (Tier 3B and 4; 669 of 3383 patients [19.8%]) per patient was 7 (3-15) (mean, 10.1; maximum, 52).

### Survival Association Between Harborage of Actionable Biomarkers

**Figure 2.**
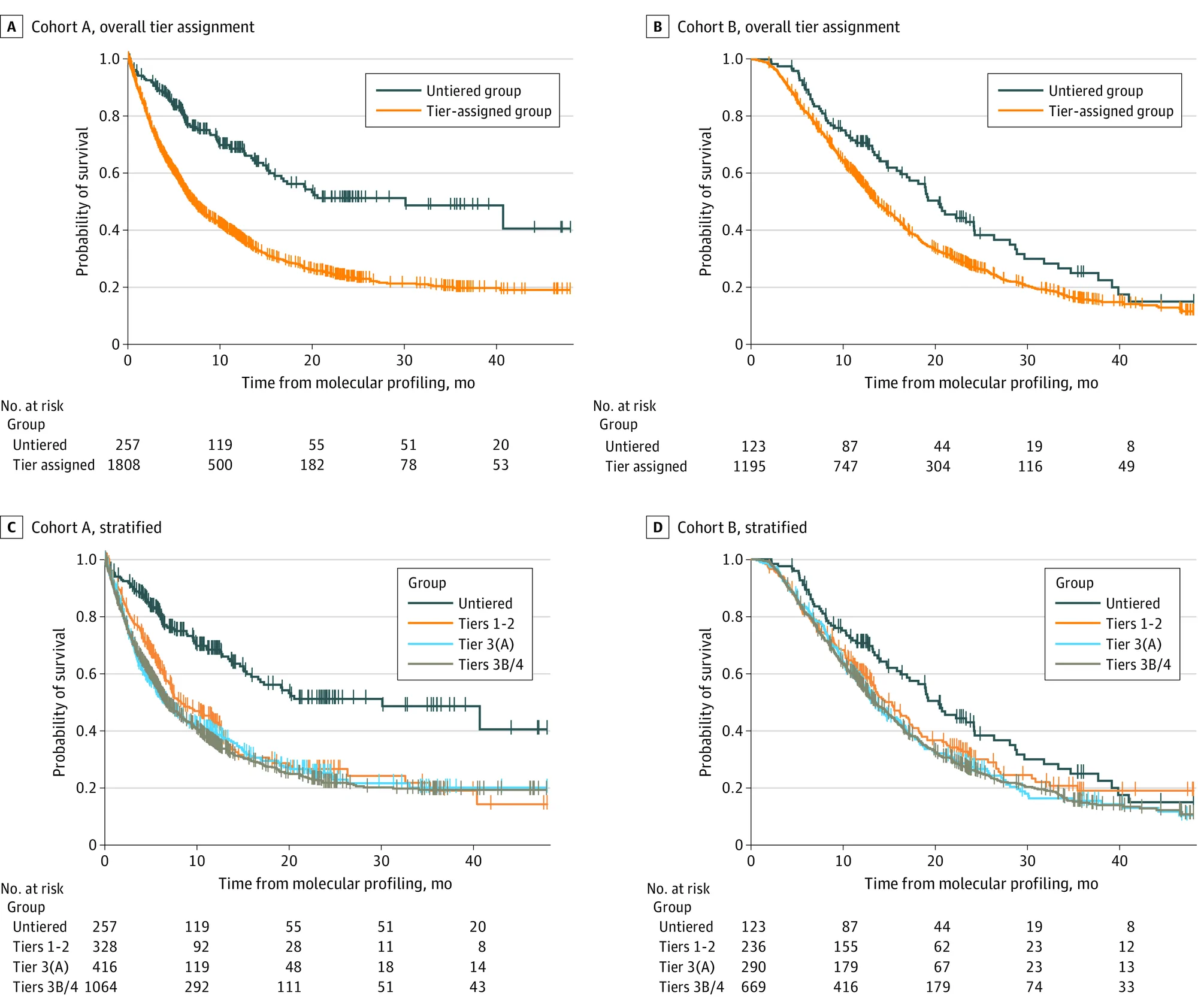
Kaplan-Meier Curves Among Distinct Prognostic Groups Stratified by Matching Status in Patients With Advanced Cancers Undergoing Genomic Profiling This figure presents the analysis of overall survival in cohorts A and B from the time of genomic profiling, by whether the genomic profiles were matched to any TOPOGRAPH (Therapy-Oriented Precision Oncology Guidelines for Recommending Anticancer Pharmaceuticals) tier or stratified. Overall survival is measured from the date of genomic profiling. The forest plot showing adjusted hazard ratios of prognostic groups is shown in eFigure 2 in Supplement 1.

For patients who did not undergo further therapy (cohort A), carriage of tier 1 to 4 genomic alteration was associated with a statistically significant shorter OS compared to patients without a matching tier (median, 7.1 months [95% CI, 6.5-7.7 months] vs 30.1 months [95% CI, 16.6 months to not reached]; unadjusted HR, 2.39; 95% CI, 1.93-2.97; *P* < .001; Figure 2A). Similar results were obtained for patients in cohort B who received unmatched therapies, with those in tier 1 to 4 having a median OS of 13.7 months (95% CI, 12.8-14.6 months) vs 20.3 months (95% CI, 16.2-24.2 months) for the untiered group (unadjusted HR, 1.43; 95% CI, 1.13-1.82; *P* < .001; Figure 2B). No differences in survival were observed when comparing individual tiers (Figure 2C and D). The differences in OS between tiered and untiered groups remained statistically significant in cohort A after adjusting for stratified age group, ECOG Performance Status, cancer type, number of lines of prior therapy, and Charlson Comorbidity Index (aHR, 1.75; 95% CI, 1.38-2.21; *P* < .001), but not in cohort B (aHR, 1.21; 95% CI, 0.95-1.55; *P* = .12) (eFigure 2 in Supplement 1). Cohorts A and B showed a similar distribution of cancer types by frequency and number of prior lines of therapy (eFigures 3 and 4 in Supplement 1).

### Survival Outcomes in Patients Who Underwent Further Therapy After Profiling

Among the 1318 patients in cohort B, 382 (29.0%) received 1 or more lines of systemic therapy after genomic profiling, matched to tier 1 to 4 biomarkers, with the remainder receiving unmatched therapies. Compared to 936 patients receiving only unmatched therapy regardless of biomarker status, the OS was longer for patients who received matched therapies (median, 13.4 months [95% CI, 12.7-14.7 months] vs 16.3 months [95% CI, 13.8-18.4 months]; aHR, 0.76; 95% CI, 0.65-0.88; *P* < .001; eFigure 5 in Supplement 1). In 116 patients (8.9%) who received 1 or more clinically active matched therapies (tiers 1-3A), a statistically significant longer OS was observed (median, 21.2 months [95% CI, 17.1-26.8 months]; aHR, 0.54; 95% CI, 0.41-0.71, *P* < .001; eFigure 5 in Supplement 1). In contrast, 266 patients who received an investigational-matched therapy (tier 3B and 4) had a median OS of 14.2 months (95% CI, 12.7-16.8 months; aHR, 0.86; 95% CI, 0.72-1.02; *P* = .09). In 21 patients who received a therapy classified as clinically inactive (tier R2), the median OS was 9.7 months (95% CI, 5.3-17.1 months; aHR, 0.89; 95% CI, 0.50-1.60; *P* = .70).

### Estimating Treatment Effect of Matched Therapy Within Tier Groups in Cohort B

**Figure 3.**
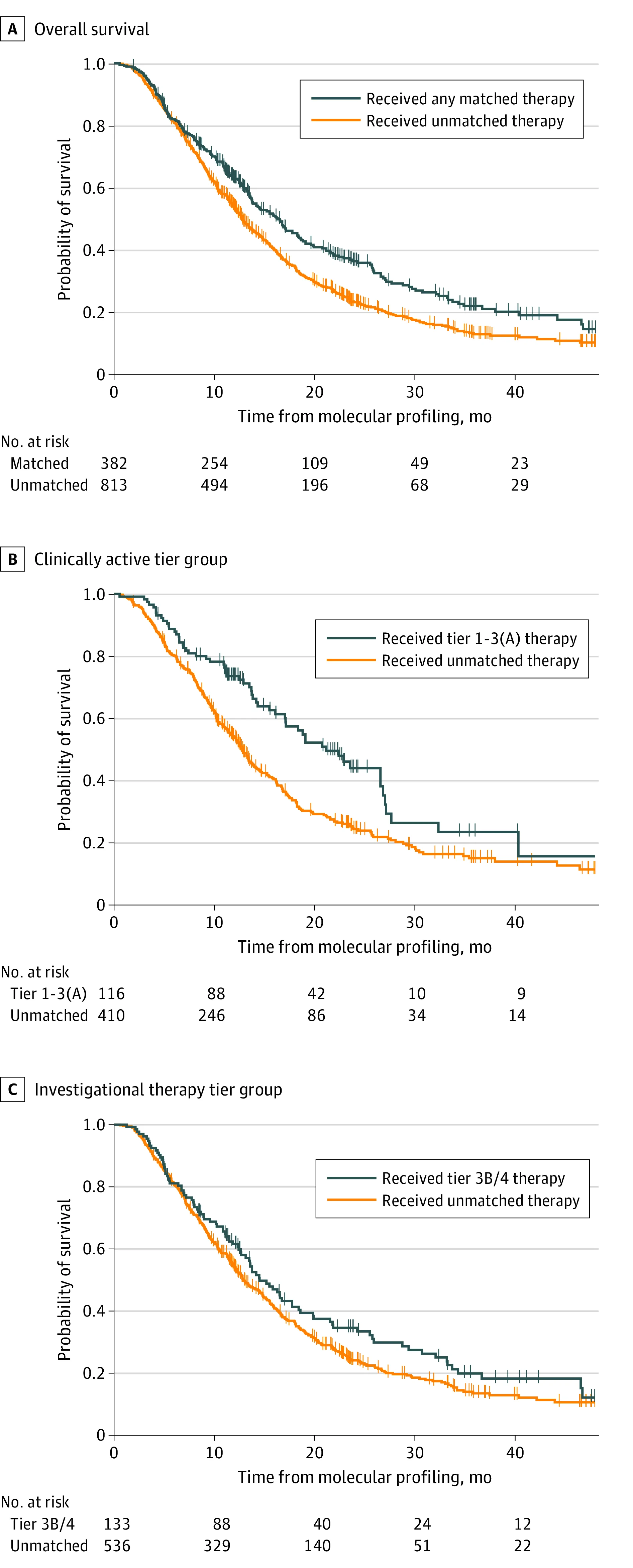
Kaplan-Meier Curves Among the Tier-Matched Analysis in Cohort B Comparing Survival Outcomes A, Difference in overall survival between the matched (tiers 1-4) and unmatched therapy groups (adjusted hazard ratio, 0.78; 95% CI, 0.66-0.91; *P* < .001). B, Analysis of patients whose highest genomically matched TOPOGRAPH (Therapy-Oriented Precision Oncology Guidelines for Recommending Anticancer Pharmaceuticals) tier was in the clinically active group (tiers 1-3) (adjusted hazard ratio, 0.60; 95% CI, 0.44-0.82; *P* < .001). C, Analysis of the investigational therapy tier group (tiers 3B/4) (adjusted hazard ratio, 1.04; 95% CI, 0.84-1.29; *P* = .71).

For those with tier 1 to 4 genomic profiles, 382 patients who received matched therapies had a statistically significant longer OS over the 813 patients who received unmatched therapy (median, 16.3 months [95% CI, 13.8-18.4 months] vs 12.8 months [95% CI, 12.1-13.8 months]; aHR, 0.78; 95% CI, 0.66-0.91; *P* = .002; Figure 3A). In the group of patients carrying a biomarker associated with at least 1 tier 1 to 3(A) therapy, the 116 patients who received a matched therapy had statistically significant longer OS than the 410 patients who received unmatched therapy (21.2 months [95% CI, 17.1-26.8 months] vs 12.8 months [95% CI, 11.7-13.9 months]; aHR, 0.60; 95% CI, 0.44-0.82; *P* = .001; Figure 3B). The magnitude of improvement in OS was comparable for receipt of matched vs unmatched therapies between tiers 1 to 2 (drugs approved by regulatory agencies) and tier 3 findings (eFigure 6 in Supplement 1). Among the 64 patients who received tier 1 to 2 matched therapies, median OS was 22.4 months (95% CI, 16.1-26.8 months) vs 13.2 months (95% CI, 10.9-15.3 months) for the 172 patients who received therapies not matched to tiers 1 to 2 (aHR 0.74; 95% CI, 0.48-1.14; *P* = .17). In the tier 3 group, matched therapy was associated with a median OS of 18.8 months (95% CI, 12.5-40.3 months) vs 12.8 months (95% CI, 11.5-14.6 months) among patients who received unmatched therapies (aHR, 0.53; 95% CI, 0.31-0.90; *P* = .02; Figure 3C).

**Figure 4.**
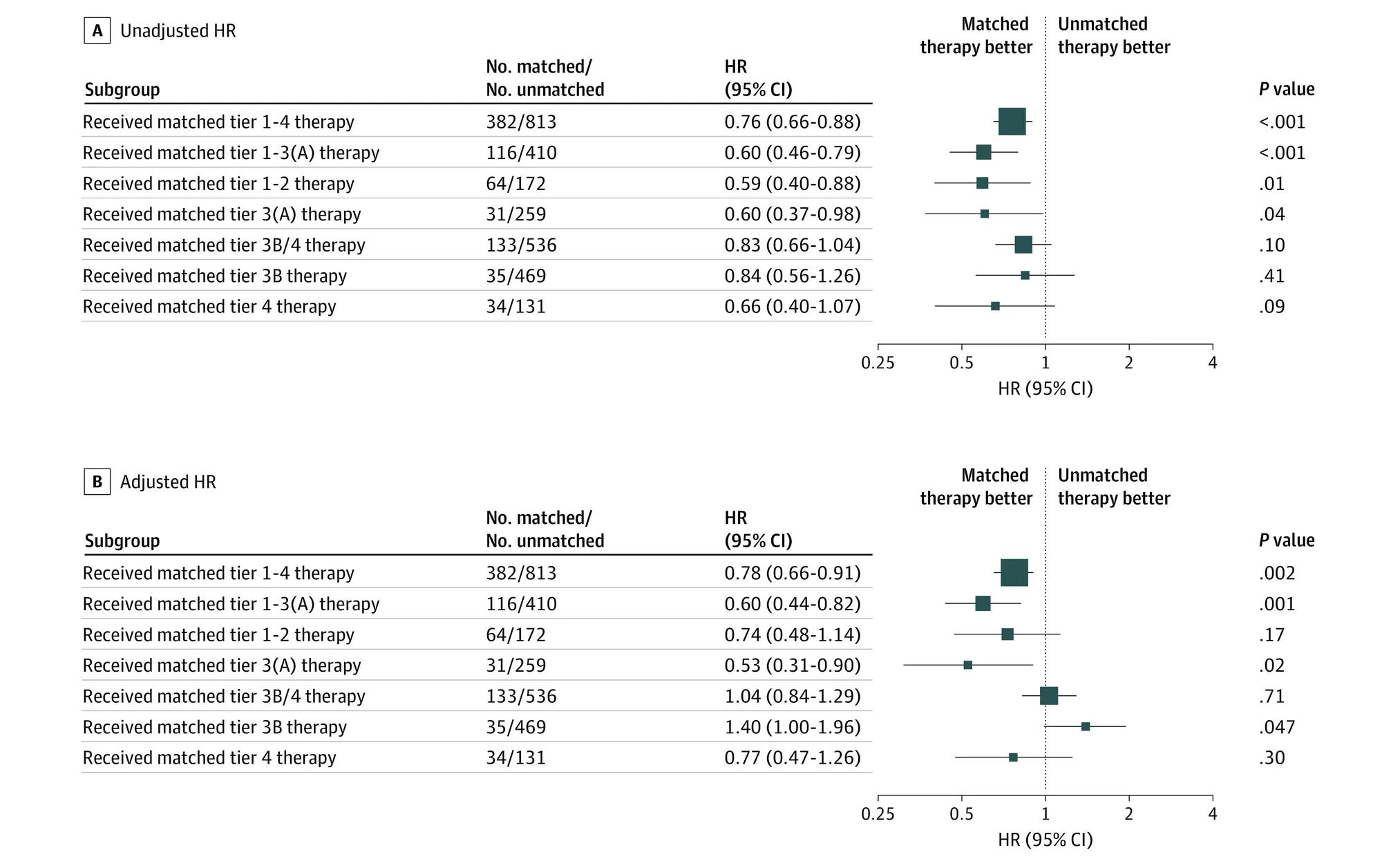
Forest Plots Showing the Unadjusted and Adjusted Hazard Ratios (HRs) from the Tier-Matched Analysis in Cohort B, Comparing Time-to-Matched Therapy With Time-to-Unmatched Therapy in Regression Models HRs were adjusted for the time to initiation of the most active subsequent therapies, age, Eastern Cooperative Oncology Group Performance Status at consent, cancer type, prior lines of therapy, and whether the patient had previously received a therapy that matched the corresponding TOPOGRAPH (Therapy-Oriented Precision Oncology Guidelines for Recommending Anticancer Pharmaceuticals) tier or tier group.

For patients with investigational tier biomarkers (tiers 3B and 4), there was no statistically significant difference in OS among the 133 patients who received matched treatments compared to the group who received only unmatched therapy after adjusting time to therapy in a time-dependent Cox regression model (median, 14.5 months [95% CI, 12.6-18.4 months] vs 12.8 months [95% CI, 12.0-14.7 months]; aHR, 1.04; 95% CI, 0.84-1.29; *P* = .71; Figure 4). Notably, there was not a statistically significant longer OS among patients who received matching therapies in tier 3B, compared with those who received unmatched therapy, based on genomic variants alone, with supporting evidence obtained only in noncognate histotypes (13.6 months [95% CI, 8.0-16.8 months] vs 12.5 months [95% CI, 11.3-13.5 months]; aHR 1.40; 95% CI, 1.00-1.96; *P* = .047; eFigure 6 in Supplement 1). Notably, there was statistically significant longer delay from genomic profiling to matched tier 3B therapy among patients who received matched therapy compared to those receiving unmatched therapy (median [IQR], 167 [59-310] days vs 75 [27-174] days; *P* = .01; eTable 3 in Supplement 1). For tier 4 (preclinical or early clinical evidence), median OS was 21.8 months (95% CI, 14.5-36.7 months) vs 16.2 months (95% CI, 12.8-19.1 months) (aHR, 0.77; 95% CI, 0.47-1.26; *P* = .30; eFigure 6 in Supplement 1). A consistent, tier-based OS difference was observed in (1) an exploratory tier-matched analysis selecting only the same therapies in both matched and unmatched groups (eFigure 7 in Supplement 1), (2) sensitivity analyses accounting for patients who died before receiving profiling results (eFigures 8 and 9 in Supplement 1), and (3) analyses using updated TOPOGRAPH tiering knowledge base (eFigure 10 in Supplement 1).

## Discussion

A clear definition of therapy matching can guide oncologists in selecting among multiple biomarker-dependent treatments for patients with advanced cancers. Through the MoST program, we observed tier-dependent differences in both natural history and potential treatment effect based on genomic profiling. Allowing for the observational nature of this study, these results support implementing a strength-of-recommendation tiering framework as a strategy for rationally prioritizing additional lines of genomically informed therapy in heavily treated patients with solid malignant tumors, providing a pragmatic strategy for structured decision-making.

We observed a 40% reduction in the risk of death associated with the receipt of genomic matched therapy in the clinically active tier group (tiers 1-3A) after accounting for confounders and time-related biases. Assignment to this group was based on supportive evidence from diverse, prospective biomarker-linked trials with at least evidence from phase 2 (or equivalent) trials.^27^ Conversely, we found no OS difference in patients who received treatment matched to the investigational therapy tier groups overall (tiers 3B and 4). The observation of lack of survival difference regarding drug repurposing (tier 3B) was particularly noteworthy, as inferring treatments based on biomarkers alone from evidence extrapolated from a noncognate cancer type is commonly used in the absence of histotype-specific trials or regulatory approvals. Off-label repurposing of drugs should be discouraged outside of clinical trials, or at least prompt a discussion with the patient about options not informed by genomics to avoid ineffective treatment. The present results corroborate international guidelines.^29,30^

Through the MoST program, 37.5% of patients profiled carried a tier 1 to 3(A) biomarker, although only a minority (3.4%) received a matched therapy. While clinical factors could affect uptake of a therapy, part of this disparity could be attributed to drug access patterns in Australia, warranting services research to identify barriers that preclude treatment access. Notably, the magnitude of OS benefit for unapproved drugs in tier 3 was similar to approved therapies (tiers 1-2), which could advocate for expanded eligibility to access relevant biomarker-dependent trials to allow better equitable access. Earlier genomic profiling in the cancer journey may also allow patients timely access to appropriate investigational matched therapies.

For tier 4 matched therapies, the observed magnitude of OS differences corroborated the trend where modern phase 1 trials have seen higher response rates in recent years.^31^ Overall, this study reinforced the importance of ongoing trials and innovative precision medicine platforms to efficiently support or discontinue biomarker-drug pairs to refine the continuously changing evidence landscape in precision oncology, as recently illustrated in the Molecular Analysis for Therapy Choice (MATCH) trial.^32^ Continuing investment in platform trials complements randomized trials in rare cancers (70% of the MoST cohort), which account for more than 40% of cancer-related deaths in Australia.^33^

### Limitations

This study has highlighted the value of a tier-based matching framework, although comparison with other level-of-evidence systems, including the European Society for Medical Oncology Scale for Clinical Actionability of Molecular Targets,^34^ would be necessary to understand the optimal matching strategy in future studies. This study also did not include patients who died before their genomic results were available, although a sensitivity analysis showed the magnitude of OS difference remained consistent with the primary analysis. Further examining tiering strategies should also incorporate nuanced interactions between evolving standards of care, genomic variants, tumor histology, and treatment histories to support standardized decision-making for prioritizing therapies and clinical trials.

## Conclusions

In this cohort study of patients with solid tumors, analysis of the MoST screening program demonstrates that genomic matching is associated with observable differences in overall survival when underpinned by prospective clinical trial evidence. These findings also support the use of evidence-based frameworks for prioritizing therapeutic stratification in advanced cancers. Overcoming barriers to drug access and elucidating the interplay between molecular and histotypic response determinants, particularly in rare cancers, necessitates international collaboration. By pooling data to power expanded cohort studies, the global oncology community can refine recommendation standards and maximize the clinical utility of comprehensive genomic profiling.

## Notes

Supplement 1.
eMethods
**eFigure 1.** Oncoplot showing histotype, frequency of genomic alterations and co-mutations, tumor mutational burden, and TOPOGRAPH tiers in 3,383 patients across both cohorts **eFigure 2.** Forest plot showing adjusted HR distinct prognostic groups stratified by potential matching status **eFigure 3.** Distinct prognostic groups stratified by matching status in patients with advanced cancers undergoing genomic profiling **eFigure 4. **Scatter plot showing concordance of frequency in patients with a tier-assigned genomic profile between Cohorts A and B **eFigure 5.** Overall survival in Cohort B by matched therapy and tiers **eFigure 6.** Tier-matched analysis in Cohort B showing the difference in survival between patients within the same tier group who received genomically matched, versus unmatched, therapies **eFigure 7. **Exploratory tier-matched analysis selecting only the same therapies in both matched and unmatched group **eFigure 8. **Sensitivity analysis of survival from Time of Consent, incorporating patients who died prior to receiving genomic results **eFigure 9. **Sensitivity analysis of tier-matched analysis from time of consent, incorporating patients who died prior to receiving genomic results **eFigure 10. **Tier-matched sensitivity analysis comparing survival for geomically matched versus unmatched therapies using TOPOGRAPH version 20251018 **eFigure 11. **Landmark analyses of survival between matched, unmatched, and untreated groups after genomic profiling **eTable 1. **Characteristics of the solid tumor cohort in the MoST study **eTable 2. **Highest actionable TOPOGRAPH tiers in patients with at least one potential therapy matched to the genomic profile **eTable 3. **Median time-to-most active therapy in Cohort B **eTable 4. **Differences in gene coverage of various genomic profiling panels used in the MoST study **eTable 5. **Drug classes of matched and unmatched most active therapies in Cohort B

Supplement 2. **Nonauthor Collaborators. **The Australian Molecular Screening and Therapeutic program investigators and contributors

Supplement 3.
Data Sharing Statement
