# Supplementary material for: Genomic Therapy Matching in Rare and Refractory Cancers
Source: JAMA Oncol. 2026 Mar 5;12(5):458–67. doi: 10.1001/jamaoncol.2026.0127 (PMC12964253; doi:10.1001/jamaoncol.2026.0127)
Supplement: Supplement 1. — eMethods eFigure 1. Oncoplot showing histotype, frequency of genomic alterations and co-mutations, tumor mutational burden, and TOPOGRAPH tiers in 3,383 patients across both cohorts eFigure 2. Forest plot showing adjusted HR distinct prognostic groups stratified by potential matching status eFigure 3. Distinct prognostic groups stratified by matching status in patients with advanced cancers undergoing genomic profiling eFigure 4. Scatter plot showing concordance of frequency in patients with a tier-assigned genomic profile between Cohorts A and B eFigure 5. Overall survival in Cohort B by matched therapy and tiers eFigure 6. Tier-matched analysis in Cohort B showing the difference in survival between patients within the same tier group who received genomically matched, versus unmatched, therapies eFigure 7. Exploratory tier-matched analysis selecting only the same therapies in both matched and unmatched group eFigure 8. Sensitivity analysis of survival from Time of Consent, incorporating patients who died prior to receiving genomic results eFigure 9. Sensitivity analysis of tier-matched analysis from time of consent, incorporating patients who died prior to receiving genomic results eFigure 10. Tier-matched sensitivity analysis comparing survival for geomically matched versus unmatched therapies using TOPOGRAPH version 20251018 eFigure 11. Landmark analyses of survival between matched, unmatched, and untreated groups after genomic profiling eTable 1. Characteristics of the solid tumor cohort in the MoST study eTable 2. Highest actionable TOPOGRAPH tiers in patients with at least one potential therapy matched to the genomic profile eTable 3. Median time-to-most active therapy in Cohort B eTable 4. Differences in gene coverage of various genomic profiling panels used in the MoST study eTable 5. Drug classes of matched and unmatched most active therapies in Cohort B [file jamaoncol-e260127-s001.pdf]

Supplemental Online Content

Lin FP, Thavaneswaran S, Grady JP, et al. Genomic therapy matching in rare and refractory cancers. *JAMA Oncol*. Published online March 5, 2026.
doi:10.1001/jamaoncol.2026.0127

eMethods.
eFigure 1. Oncoplot showing histotype, frequency of genomic alterations and co-mutations, tumor mutational burden, and TOPOGRAPH tiers in 3,383 patients across both cohorts
eFigure 2. Forest plot showing adjusted HR distinct prognostic groups stratified by potential matching status
eFigure 3. Distinct prognostic groups stratified by matching status in patients with advanced cancers undergoing genomic profiling
eFigure 4. Scatter plot showing concordance of frequency in patients with a tier-assigned genomic profile between Cohorts A and B
eFigure 5. Overall survival in Cohort B by matched therapy and tiers
eFigure 6. Tier-matched analysis in Cohort B showing the difference in survival between patients within the same tier group who received genomically matched, versus unmatched, therapies
eFigure 7. Exploratory tier-matched analysis selecting only the same therapies in both matched and unmatched group
eFigure 8. Sensitivity analysis of survival from Time of Consent, incorporating patients who died prior to receiving genomic results
eFigure 9. Sensitivity analysis of tier-matched analysis from time of consent, incorporating patients who died prior to receiving genomic results
eFigure 10. Tier-matched sensitivity analysis comparing survival for geomically matched versus unmatched therapies using TOPOGRAPH version 20251018
eFigure 11. Landmark analyses of survival between matched, unmatched, and untreated groups after genomic profiling
eTable 1. Characteristics of the solid tumor cohort in the MoST study
eTable 2. Highest actionable TOPOGRAPH tiers in patients with at least one potential therapy matched to the genomic profile
eTable 3. Median time-to-most active therapy in Cohort B
eTable 4. Differences in gene coverage of various genomic profiling panels used in the MoST study
eTable 5. Drug classes of matched and unmatched most active therapies in Cohort B

This supplemental material has been provided by the authors to give readers additional information about their work.

eMethods.

Study protocol and oversight

This cohort study was conducted within the Molecular Screening and Therapeutics (MoST) program (Australian New Zealand Clinical Trials Registry ID: ACTRN12616000908437), a national precision oncology initiative [28]. The study protocol was approved by the St Vincent's Hospital Human Research Ethics Committee (HREC/16/SVH/23), and all participants provided written informed consent prior to enrollment. The MoST program also incorporates independent Phase 2 interventional trial modules (substudies), to which small subset of patients with matching molecular criteria may be eligible to enroll, of which the results are reported separately. This analysis was conducted in accordance with the MoST Framework and the Pan-Cancer Subprogram appendix (Version 13.0).

Study cohort

Following genomic profiling, the study population was stratified into two cohorts based on the administration of subsequent systemic therapy. Cohort A comprised patients who received no further systemic therapy after the return of genomic results. Cohort B comprised patients who received at least one line of systemic therapy following successful genomic profiling. This stratification was performed to facilitate a meaningful analysis of treatment outcomes, as significant heterogeneity in baseline demographics and survival characteristics was observed across the entire screened population. (eFigure 11).

Genomic profiling

Genomic profiling was performed on archival formalin-fixed, paraffin-embedded (FFPE) diagnostic material. Next-generation multigene panels, primarily the Illumina TruSight Oncology panels (TST170 and TSO500) and the FoundationOne CDx assay, were used. Identified genomic variants were subsequently reviewed by a molecular tumor board (MTB) at weekly meetings (fortnightly at Western Australian sites). This review formed the basis for clinical interpretation and the identification of potential targeted therapies. The differences in gene coverage of various genomic profiling panels used in the MoST study is shown in eTable 4.

Tier assignment

© 2026 Lin FP et al. JAMA Oncol.

To prevent subjective bias, the potential highest tier of therapy for each patient's genomic profile was assigned using a computer algorithm [31]. For patients in Cohort B, the overall tier of the therapy actually received after genomic profiling was determined according to a pre-defined hierarchy for the 'most active' therapy. This was defined as: (1) the therapy with the highest TOPOGRAPH tier among the therapies received subsequent to genomic profiling; (2) for multiple therapies genomically matched to the same tier, the tier of the earliest therapy administered; or (3) classified as unmatched if the therapy had no corresponding genomic biomarker. For each patient with a valid genomic profile, a complete list of potential biomarker-linked therapies (both single drug or drug combinations) with potential tiers was algorithmically inferred using Precision Oncology Treatment and Trials Recommender (POTTR), a forward-chaining inference engine that integrates purposely-built ontologies and TOPOGRAPH knowledge base to exhaustively reason and prioritize all possible biomarker-linked treatments given a clinicogenomic profile [31]. The following tier ranking order was applied for prioritising therapy lists: 1> 1B> 2> 3> 3B> 4> R2.

The TOPOGRAPH knowledge (version AU: 20220828) was applied to the genomic profiles of the entire cohort. The knowledge base comprised 282 biomarkers (221 gene and protein, 49 combinations, and 12 complex biomarkers) and 1743 alterations (including 903 simple genomic variants, 331 variant groups, 120 gene amplification and deletions, 228 fusions and rearrangements, and 5 splice variants) in 158 cancer types (27 common, 15 less common, and 122 rare cancers). A total of 576 therapies (drug and/or drug combinations) were stratified into clinically active (69 Tier 1, 32 Tier 1B, 90 Tier 2, 170 Tier 3), investigational (369 Tier 4), and inactive tier groups (17 Tier R1, 180 Tier R2), in addition to individual Tiers (see Figure 1B for definition). [29]

Classification of source of therapy (SOC, non-SOC, or clinical trial) was algorithmically searched against the eviQ database https://eviq.org.au.

Determining tier of biomarker-linked therapies received

To determine which therapy was genomically matched in a patient's treatment history, an entry from the treatment record (drug and drug regimen) was searched against the potential list of biomarker-linked therapies inferred by POTTR. An overall tier of therapy was assigned if any of the following was satisfied: (1) complete match of the drug regimen name; (2) complete match of the drug class combination; (3) partial match to a drug class if the therapy

© 2026 Lin FP et al. JAMA Oncol.

contains a combination of more than one drug (Figure 1C); otherwise the therapy was considered untiered (unmatched). The tiers assigned to drug classes based on the biomarker profile are summarized in eTable 5.

Outcome measures

The primary outcome was overall survival (OS), defined as the time from receipt of molecular profiling results to death from any cause. Participants underwent follow-up assessments at 6 and 12 months after molecular profiling, then annually for up to 5 years. For the OS analysis, data were censored at 20 July 2022, with patients of unknown survival status censored at their last documented follow-up date. Secondary outcomes included the frequency and type of actionable molecular alterations identified, the proportion of patients who received genomically matched therapy, and the molecular profiling turnaround time, measured in days from sample receipt to report finalization.

A proportion of participants who underwent screening participated in the therapeutic substudies, which operated independently of the molecular screening program. The primary endpoints of these therapeutic substudies, which determine drug activities through treatment response based on the radiologic criteria, did not employ overall survival (OS) as the primary endpoint. The OS data reported in this study were collected through independent follow-up described above, unrelated to the interventional trials.

Statistical Analysis

To define the most active therapy as the first highest-tier treatment matched to the subsequent therapy, or if no therapies were matched to a biomarker, the first therapy subsequent to genomic profiling was designated the most active therapy. The tier associated with most active therapy is used to determine the overall tier for each patient, allowing per patient assessment on the premise that the receipt of this therapy would be the most important determinant (biologically, as the activity of rationally targeted therapy; and clinically, as earlier therapies, are postulated to be more active) contributing to survival outcomes.

To more precisely measure the effect of therapy matching, two additional tier-matched analyses were conducted in (1) a subset of patients who only received one line of therapy after genomic profiling, and (2) a drug-matched analysis in which the treated group was

© 2026 Lin FP et al. JAMA Oncol.

compared to a pool of patients who had the same medication administered but did not possess the genomic biomarker as the control.

A post hoc exploratory subgroup analysis was conducted to examine how the treatment effect of genomic therapy matching may be modified by cancer type, biomarker, and drug therapies subgroups. All subgroups of genomic biomarkers (presence or absence of genomic alteration) and drug classes that had a minimum of three patients in the matched therapy group were analyzed. Partial likelihood test was applied to determine the significance of the interaction of the interaction term at the threshold of 0.05, between OS matched therapy and each subgroup. Bonferroni correction was employed to limit the family-wise type 1 error rate arising from the multiple comparisons of the main effects.

Software

The R *survival* package was used for survival analysis. R statistical environment version 4.0.5 was used for both descriptive and regression analyses. The TOPOGRAPH database can be accessed at https://topograph.info. The Precision Oncology Treatment and Trials Recommender (POTTR) tool, which includes the associated precision cancer type and drug ontologies, is available as open-source software on GitHub at https://github.com/fpylin/POTTR.

© 2026 Lin FP et al. JAMA Oncol.

List of MoST investigators and contributors

Principal investigators (MoST framework): David Thomas, Garvan Institute of Medical Research, NSW; John Simes, NHMRC Clinical Trials Centre, University of Sydney, NSW; Mandy Ballinger, Garvan Institute of Medical Research, NSW; Michail Charakidis, Royal Darwin Hospital, NT; Paul Craft, Canberra Hospital, ACT; Jayesh Desai, Peter MacCallum Cancer Centre, VIC; Brett Gordon Hughes, The Prince Charles Hospital, QLD; Malinda Itchins, Royal North Shore Hospital, NSW; Chris Karapetis, Flinders Medical Centre, SA; Steven Lane, QIMR Berghofer and Royal Brisbane and Women's Hospital, QLD; Chee Khoon Lee, St George Hospital, NSW; Adrian Pokorny, Royal Darwin Hospital, NT; Geoffrey Peters, Canberra Hospital, ACT; Sagun Parakh, Austin Hospital, VIC; Kenneth O'Byrne, Princess Alexandra Hospital, QLD; David Ross, Royal Adelaide Hospital, SA; Katrin Sjoquist, St George Hospital, NSW; Mark Shackleton, The Alfred Hospital, VIC; Michael Millward, Linear Clinical Research, WA; Adnan Mahmood Nagrial, Westmead Hospital, NSW; David Goldstein, Prince of Wales Hospital, NSW; Michael Brown, Royal Adelaide Hospital, SA; Rosemary Harrup, Royal Hobart Hospital, TAS; Melissa Moore, St Vincent's Hospital, Melbourne, VIC; Maggie Moore, The Alfred Hospital, VIC; Anthony Joshua, St Vincent's Public Hospital & The Kinghorn Cancer Centre, Sydney, NSW; Peter Grimison, Chris O'Brien LifeHouse, NSW; Craig Underhill, Border Medical Oncology Research Unit, VIC.

Investigators and contributors:

New South Wales: Garvan Institute of Medical Research, NSW - Hayley Barker; Emily Collignon; Laura Conole; Mark Cowley; Jenny Gu; Elektra Hajdu; Erin Heyer; Luke Hesson; Tharindi Ip; Amelia Mifsud; Mark Pinese; Aaron O'Grady; Amy Prawira; Min Ru Qiu; Audrey Silvestri; Keith Thornton; Kelly Walwyn; Cheryll Ye; Anaiis Zaratzian. NHMRC Clinical Trials Centre, University of Sydney - Nicola Barrie; Sarah Chinchen; Michelle Cummins; Yogita Dheer; Kathleen Harwood; Enam Hoque; Sarah Finlayson; Clarencia Lie; Ian Marschner; Rachael Morton; James Murray; Kelly Nicholas; Tosin Omotoso; Sachie Pallimulla; Prapti Pandya; Isabella Richardson; Nick Ristevski; Lucille Sebastian; Hayley Thomas; Patrick Wheeler; Vicki Xie. Omico / Australian Genomic Cancer Medicine Centre - Vera Terry.

© 2026 Lin FP et al. JAMA Oncol.

South Australia: Hamish S Scott, Department of Genetics and Molecular Pathology and Centre for Cancer Biology, SA Pathology, Adelaide; Julia Dobbins, Department of Genetics and Molecular Pathology and Centre for Cancer Biology, SA Pathology, Adelaide; Anna L Brown, Department of Genetics and Molecular Pathology, SA Pathology and Centre for Cancer Biology, University of South Australia, and Adelaide Medical School, University of Adelaide, Adelaide; Alan McGovern, Department of Genetics and Molecular Pathology, SA Pathology, Adelaide, Centre for Cancer Biology, Adelaide; Centre for Cancer Biology, Adelaide - Rob King; Andreas W Schreiber. Royal Adelaide Hospital, Adelaide - Rachael Chang; Gonzalo Tapia-Rico; Melinda Whelan.

Western Australia: Timothy Humphries: School of Medicine, University of Western Australia.

© 2026 Lin FP et al. JAMA Oncol.

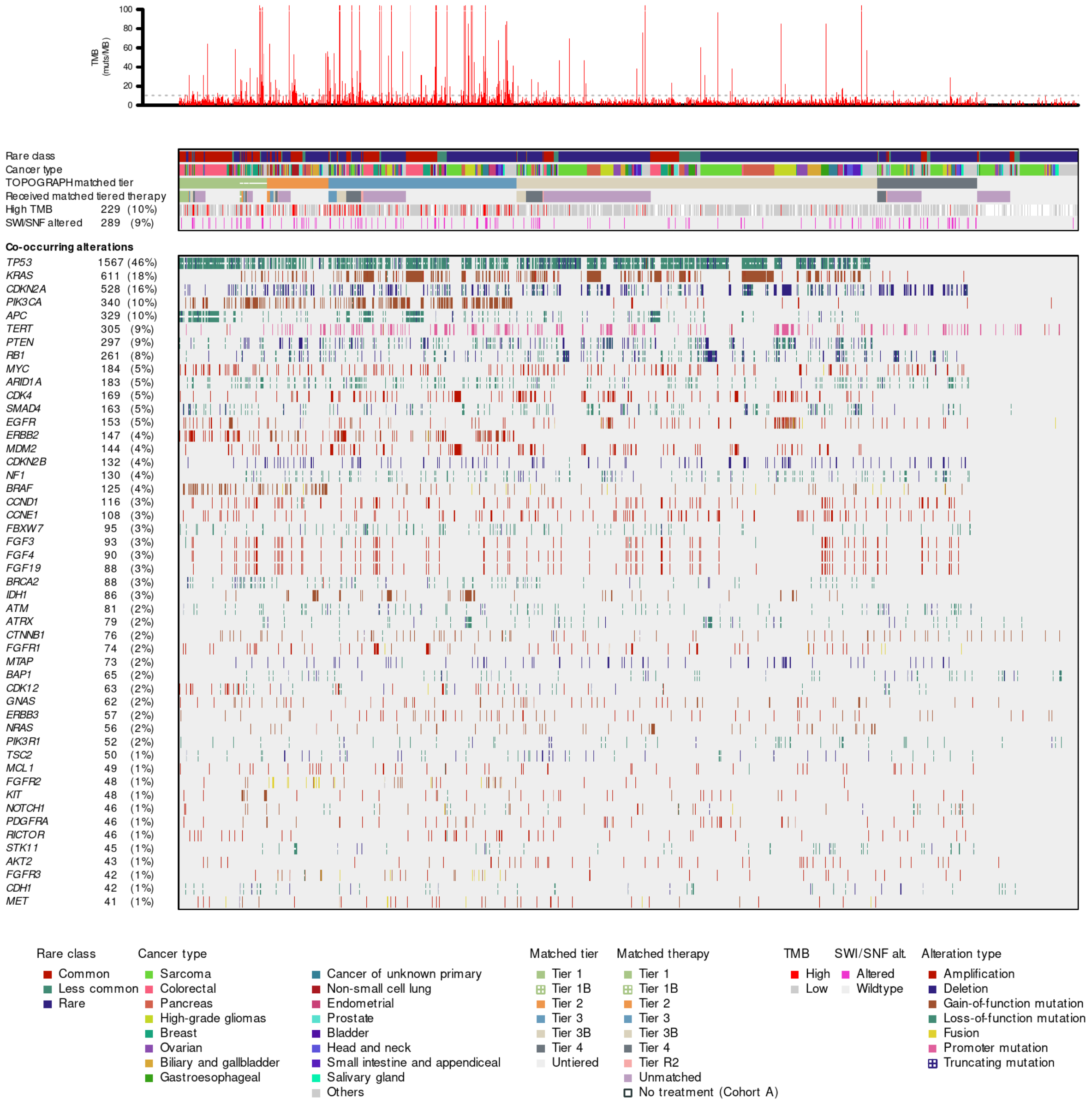

eFigure 1. Oncoplot showing histotype, frequency of genomic alterations and co-mutations, tumor mutational burden, and TOPOGRAPH tiers in 3,383 patients across both cohorts. The most frequent (≥10%) genomic alterations were *TP53* (n=1,567, 46%), *KRAS* (n=611, 18%), , including 29 *KRAS*G12C mutations across 9 cancer types *CDKN2A* (n=528, 16%), and *PIK3CA* (n=340, 10%). The median number of reportable alterations per patient after variant interpretation at the molecular tumor board (MTB) was 3 (range 0 to 27). Regarding the frequency of pan-cancer biomarkers associated with U.S. Food and Drug Administration (FDA)-approved indications, 42 solid tumors were found to have microsatellite instability

© 2026 Lin FP et al. JAMA Oncol.

(MSI). Eighteen and 9 patients had *RET* and *NTRK1-3* fusions respectively. *BRAF*^{V600} mutation was detected in 106 patients (3.1%). Tumor mutational burden (TMB) was assessable in 2,384 patients; among these, 229 patients were observed with 10 or more mutations per megabase (9.6%). This co-mutation plot illustrates the results of applying TOPOGRAPH's therapy tiering approach to map diverse tissues of origin and genomic alterations into predefined summary tiers for both therapy actionability and records. Only genes with alteration frequency ≥40 (1.2%) are shown in this plot. The ordering of the heatmap is determined by the frequency of genomic actionability (TOPOGRAPH matched tier), the actual tier of matched therapy received, and the frequency of altered genes. The most common biomarker linked to matched therapies were against alterations in *ERBB2* (n=54), *CDKN2A* (n=51), as well as high tumor mutational burden (n=45). Abbreviations: TMB: Tumor mutational burden. SWI/SNF: SWItch/Sucrose Non-Fermentable complex. Alt.: alteration. The white squares in the TMB column indicate that TMB cannot be determined.

© 2026 Lin FP et al. JAMA Oncol.

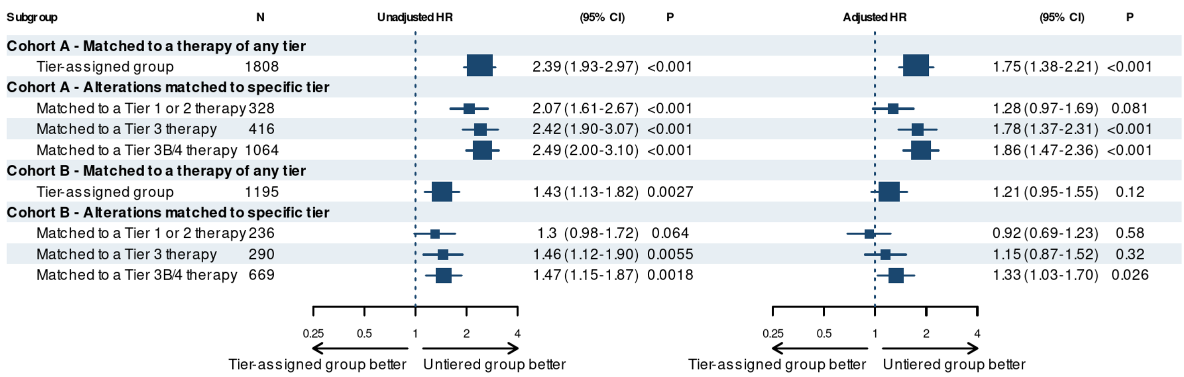

eFigure 2. Forest plot showing adjusted HR distinct prognostic groups stratified by potential matching status. Forest plot showing adjusted HR considering age, ECOG performance status at the time of consent, cancer type, and Age < 65 years, cancer type, less than one line of prior therapy, Charlson Comorbidity index.

© 2026 Lin FP et al. JAMA Oncol.

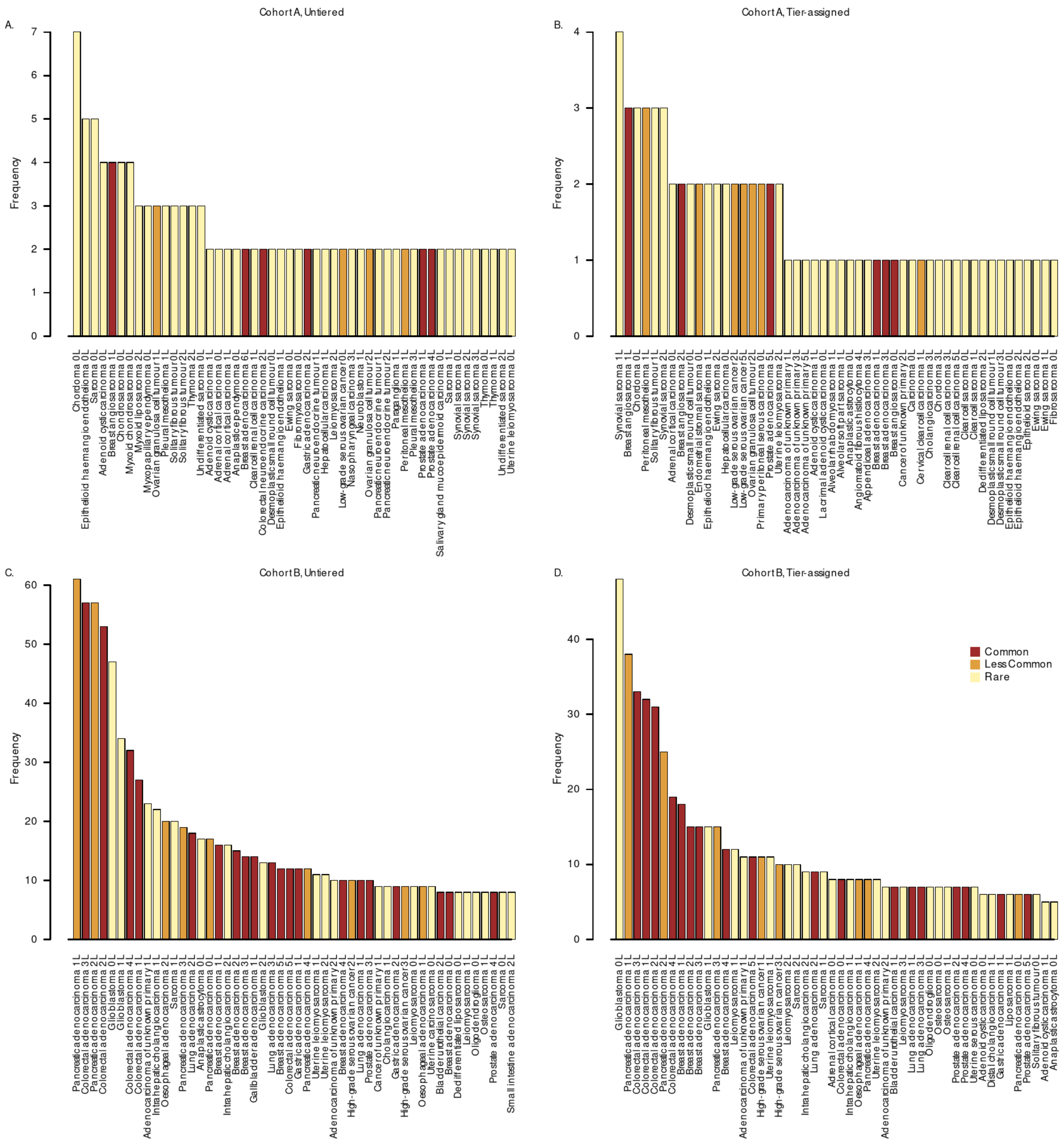

eFigure 3. **Distinct prognostic groups stratified by matching status in patients with advanced cancers undergoing genomic profiling.** Panels (A) to (D): frequency by cancer types and lines of systemic therapy prior to genomic profiling in patients Cohort A who were untiered (panel A) or tier-assigned (panel B), and in Cohort B (untiered, panel C; and tier-assigned panel D). Patients with rare cancers were more likely to reside within the untiered group (332 of 380, 87%, A and B). In contrast, the tier-assigned group consisted of poor prognostic cancers (for example, pancreatic cancer and high-grade gliomas), common cancer types, and cancers with molecular targeted therapies as SOC (e.g., colorectal cancers; C and D).

© 2026 Lin FP et al. JAMA Oncol.

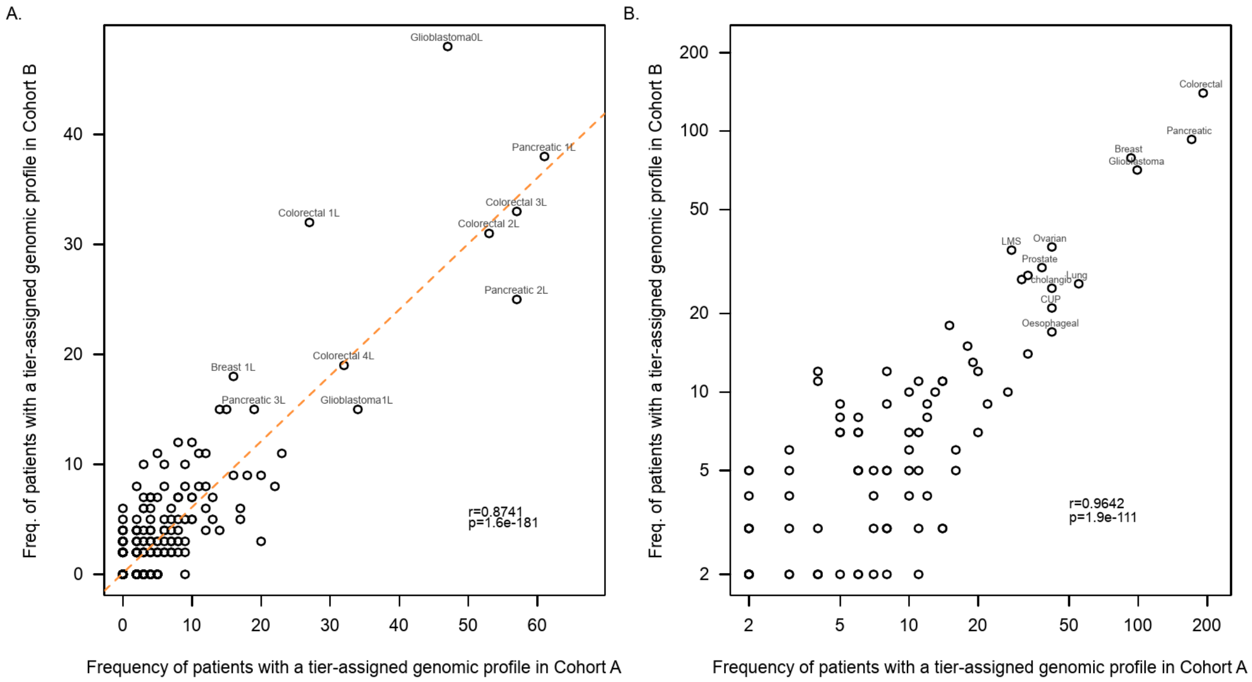

eFigure 4. Scatter plot showing concordance of frequency in patients with a tier-assigned genomic profile between Cohorts A and B by (A) Cancer types and number of prior lines of systemic therapy and (B) Cancer type.

© 2026 Lin FP et al. JAMA Oncol.

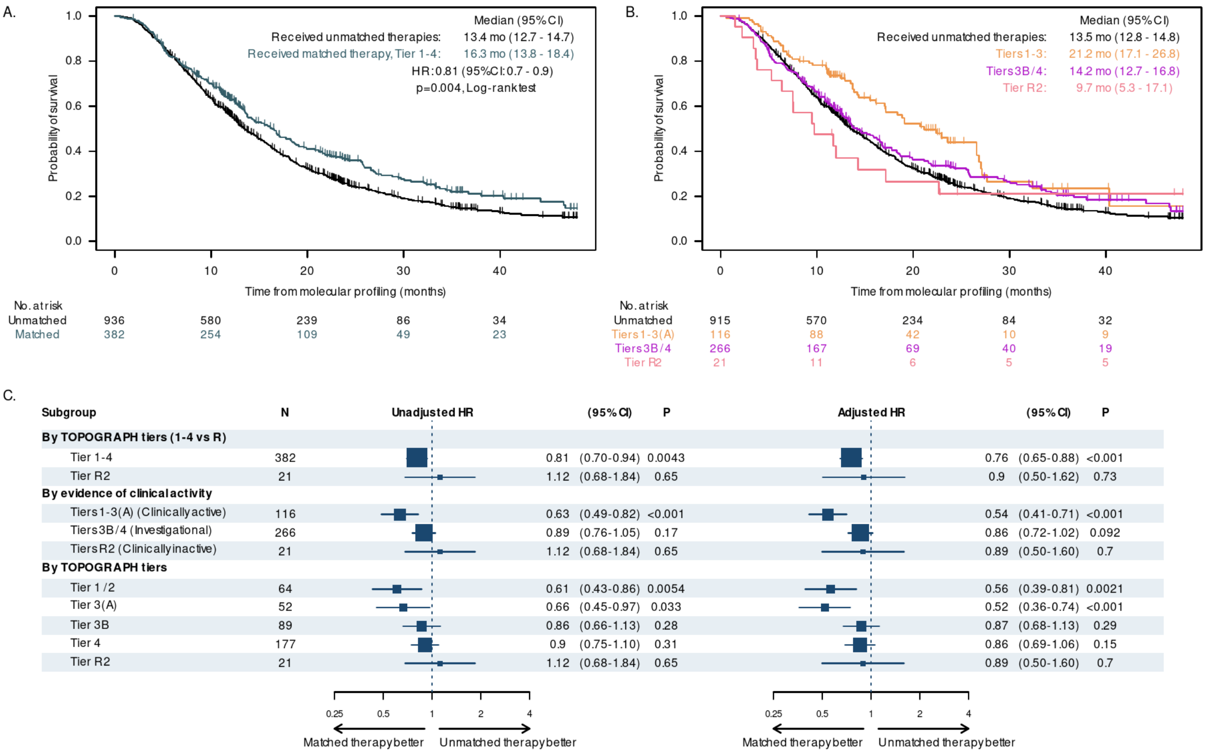

eFigure 5. Overall survival in Cohort B by (A) whether a genomically matched therapy was received (any tiers, Tiers 1-4) and (B) highest-tier matched therapy received in the clinically active (Tiers 1-3), investigational (Tier 3B/4), and inactive (Tier R2) tier groups. Panel (C) shows unadjusted and adjusted hazard ratios comparing patients receiving matched versus unmatched therapies in each stratum. This prognostic analysis compares the survival outcomes of patients who received different tiers of therapy in Cohort B against unmatched therapy. Hazard ratios were adjusted for the time-to-initiation of the most active subsequent therapies, age, ECOG performance status at the time of consent, cancer type, and whether the patient had previously received a therapy that matched the corresponding TOPOGRAPH tier.

© 2026 Lin FP et al. JAMA Oncol.

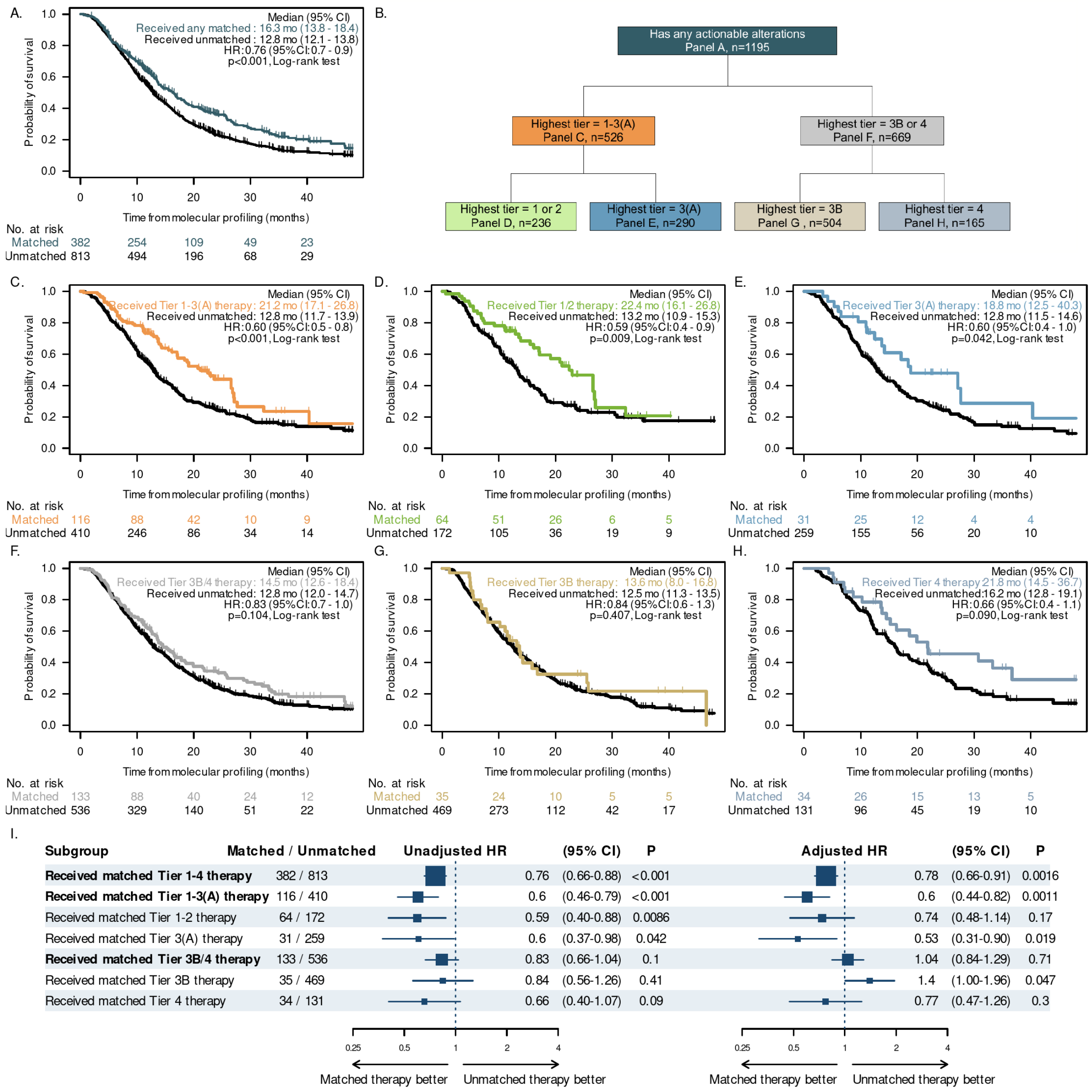

eFigure 6. Tier-matched analysis in Cohort B showing the difference in survival between patients within the same tier group who received genomically matched, versus unmatched, therapies. Panel (A) shows difference in OS between matched (Tier 1-4) versus unmatched therapy with unadjusted HR, and Panel (B) illustrates the stratification of groups by actionability in these tier-match analyses. Patients with highest genomically matched TOPOGRAPH tier in the (C) clinically active tier group (Tiers 1-3), (D) approved therapies (Tiers 1-2), (E) unapproved therapies with positive data shown in prospective trial (Tier 3), (F) investigational therapy tier group (Tier 3B and 4), (G) biomarker-matched therapy in non-cognate cancer type (Tier 3B), and (H) therapies with only preclinical or early clinical

© 2026 Lin FP et al. JAMA Oncol.

evidence (Tier 4). (I) Forest plot showing adjusted HR considering time-to-matched therapy and time-to-unmatched therapy in regression models. Hazard ratios were adjusted for the time-to-initiation of the most active subsequent therapies, age, ECOG performance status at the time of consent, cancer type, and whether the patient had previously received a therapy that matched the corresponding TOPOGRAPH tier or tier group.

© 2026 Lin FP et al. JAMA Oncol.

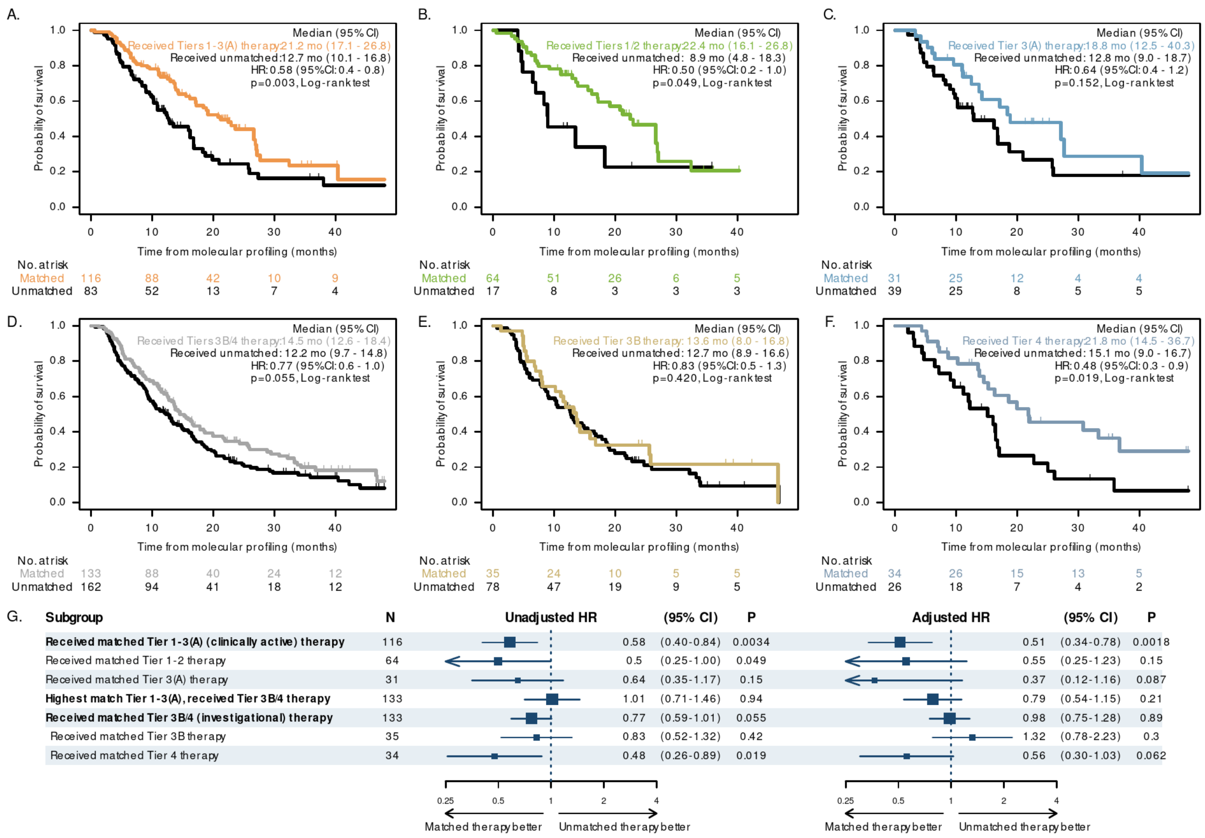

eFigure 7. Exploratory tier-matched analysis selecting only the same therapies in both matched and unmatched group. *Panels A-C*: Overall survival in patients who received a matched subsequent therapy with the highest tier matching (A) any tiers (Tiers 1-4), (B) clinically active tier group (Tiers 1-3), and (C) investigational tier group (Tiers 3B and 4). *Panels (D)-(F):* Patients who received only an unmatched therapy after genomic profiling and had the highest potential tier determined by genomics in (D) any tiers, (E) clinically active tier group (Tiers 1-3), and (F) Investigational tier group (Tiers 3B and 4). Panel (G): unadjusted and adjusted hazard ratios comparing patients receiving matched versus unmatched therapies in each subgroup. Hazard ratios were adjusted for the time-to-initiation of the most active subsequent therapies, age, ECOG performance status at the time of consent, cancer type, and whether the patient had previously received a therapy that matched the corresponding TOPOGRAPH tier or tier group.

To further illustrate the importance of therapy matching, this additional analysis to control for potential biases caused by differences in the treatment regimens used in the matched and unmatched groups was conducted. In the clinically active tier group, after restricting the control group to patients who received the same drug classes observed in the matched groups,

© 2026 Lin FP et al. JAMA Oncol.

OS remained significantly longer in 116 patients who received at least one Tier 1-3(A) therapy compared to 83 patients with the same drug-specific biomarkers but who received unmatched therapy (21.2 v 12.7 months, adjusted HR 0.51, P=0.002, Panel A). Further stratification into Tiers 1/2 and 3 revealed similar magnitude of difference in survival between matched and unmatched patients (Extended Figures 8B and 8C). Patients who received a matched treatment in the investigational therapy tier group (Tiers 3B and 4) had no significant difference in survival (matched v unmatched: 14.5 v 12.2 months, adjusted HR 0.98, P=0.89, Extended Figure 8D). No difference in OS was noted for the Tier 3B group (matched v unmatched: 13.6 v 12.7 months, adjusted HR 1.32, P=0.30, Panel E), and patients receiving matched Tier 4 therapy had a numerically longer OS (median 21.8 v 15.1 months, adjusted HR 0.56, P=0.062, Panel F).

© 2026 Lin FP et al. JAMA Oncol.

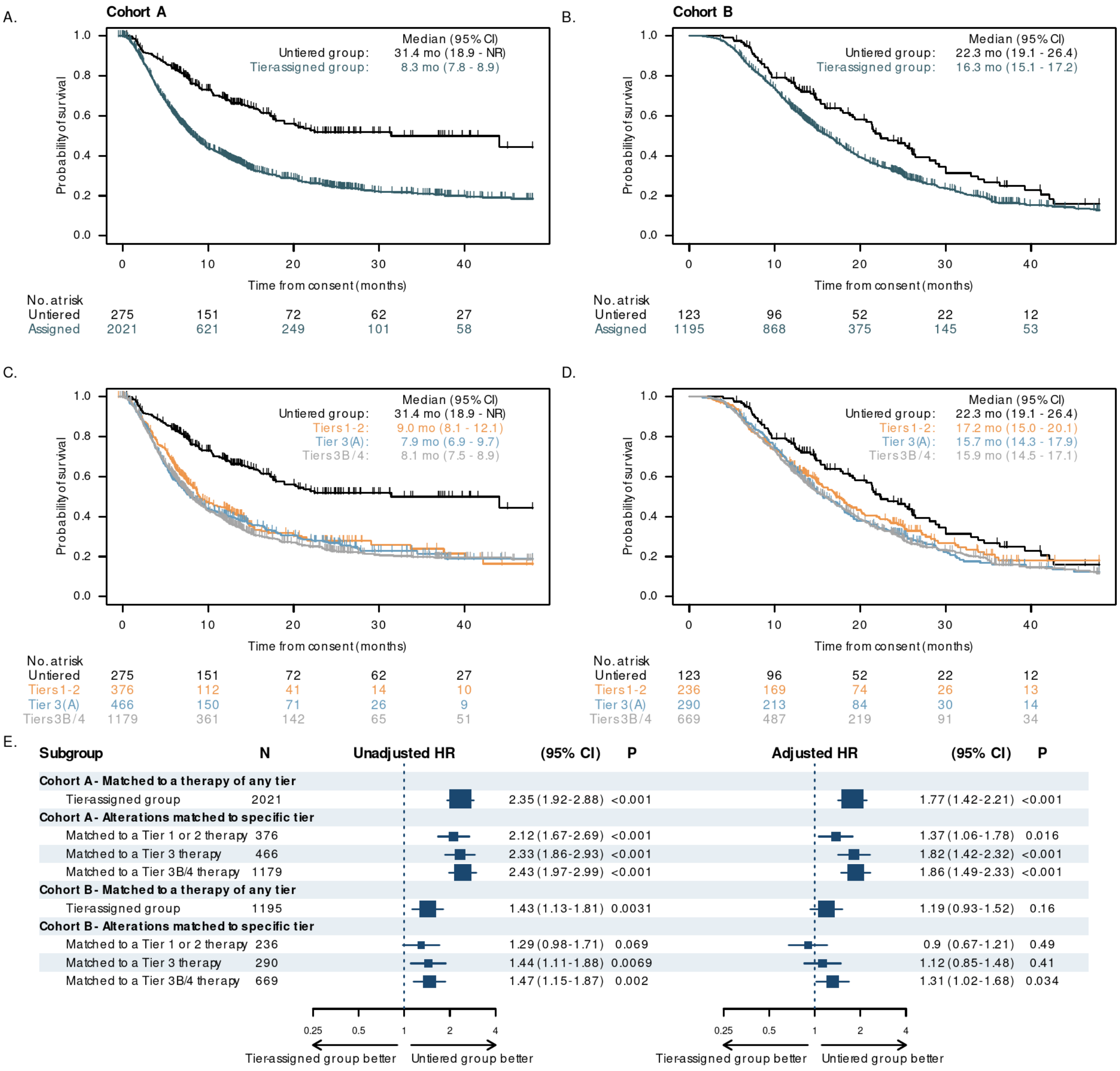

eFigure 8. Sensitivity analysis of survival from Time of Consent, incorporating patients who died prior to receiving genomic results. Prognostic groups stratified by matching status in patients with advanced cancers undergoing genomic profiling. Panels (A) to (D): Kaplan-Meier analysis of OS in Cohort A (panel A and C) and Cohort B (panels B and D) from the time of genomic profiling, by whether the genomic profiles was matched to any TOPOGRAPH tier (Tier 1-4, panels A and B) or stratified (panels C and D). OS is measured from the date of genomic profiling. (E) Forest plot showing adjusted HR considering age, ECOG performance status at the time of consent, cancer type, and Age < 65 years, cancer type, lines of prior therapy, Charlson Comorbidity index.

© 2026 Lin FP et al. JAMA Oncol.

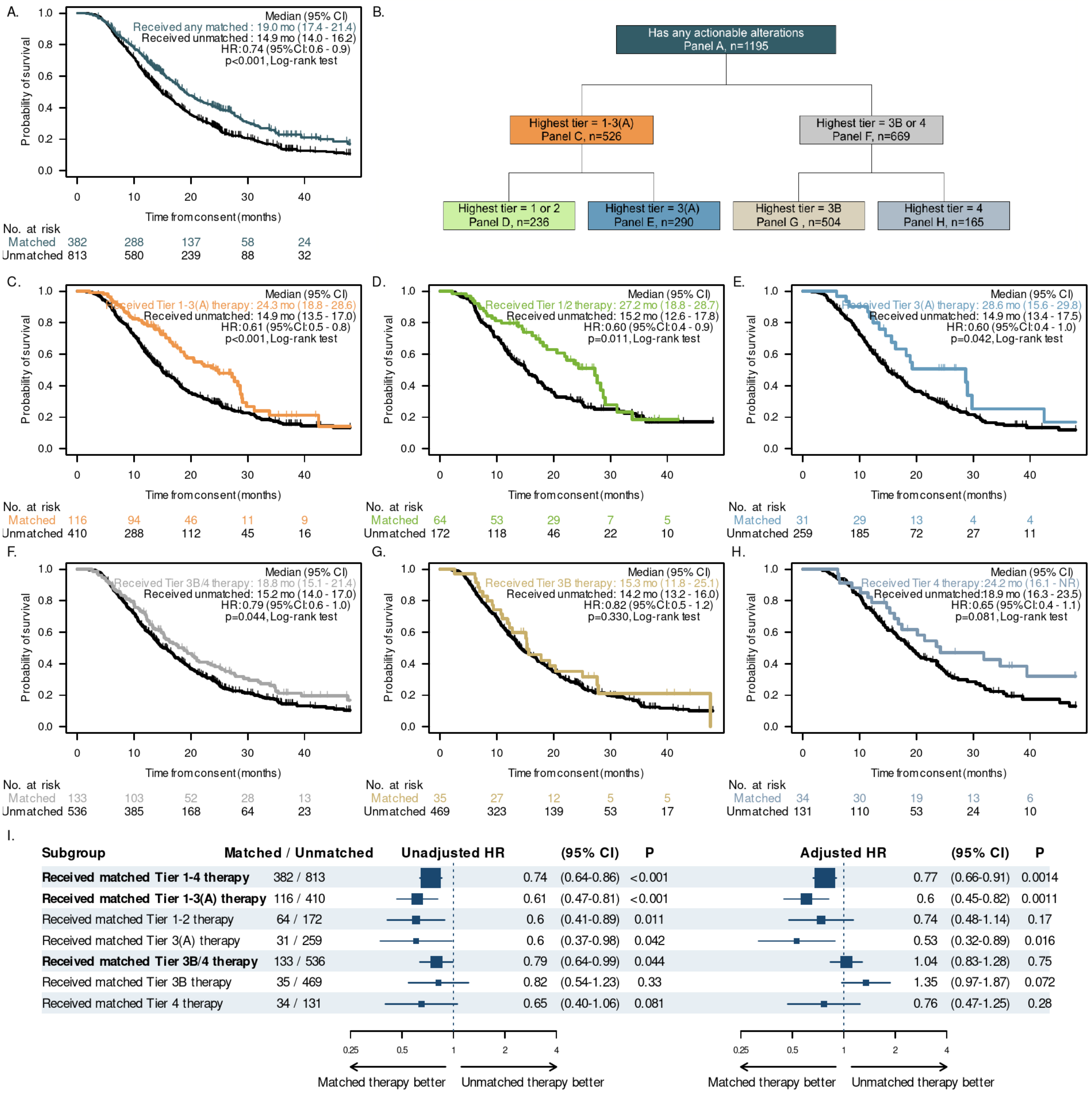

eFigure 9. Sensitivity analysis of tier-matched analysis from time of consent, incorporating patients who died prior to receiving genomic results. Similar magnitude of adjusted HR was observed in this tier-matched analysis in Cohort B showing the difference in survival between patients within the same tier group who received genomically matched, versus unmatched, therapies. Panel (A) shows difference in OS between matched (Tier 1-4) versus unmatched therapy with unadjusted HR, and Panel (B) illustrates the stratification of groups by actionability in these tier-match analyses. Patients with highest genomically matched TOPOGRAPH tier in the (C) clinically active tier group (Tiers 1-3), (D) approved therapies (Tiers 1-2), (E) unapproved therapies with positive data shown in prospective trial (Tier 3), (F) investigational therapy tier group (Tier 3B and 4), (G) biomarker-matched therapy in non-cognate cancer type (Tier 3B), and (H) therapies with only preclinical or early clinical evidence (Tier 4). (I) Forest plot showing adjusted HR considering time-to-matched therapy

and time-to-unmatched therapy in regression models. Hazard ratios were adjusted for the time-to-initiation of the most active subsequent therapies, age, ECOG performance status at the time of consent, cancer type, and whether the patient had previously received a therapy that matched the corresponding TOPOGRAPH tier or tier group.

© 2026 Lin FP et al. JAMA Oncol.

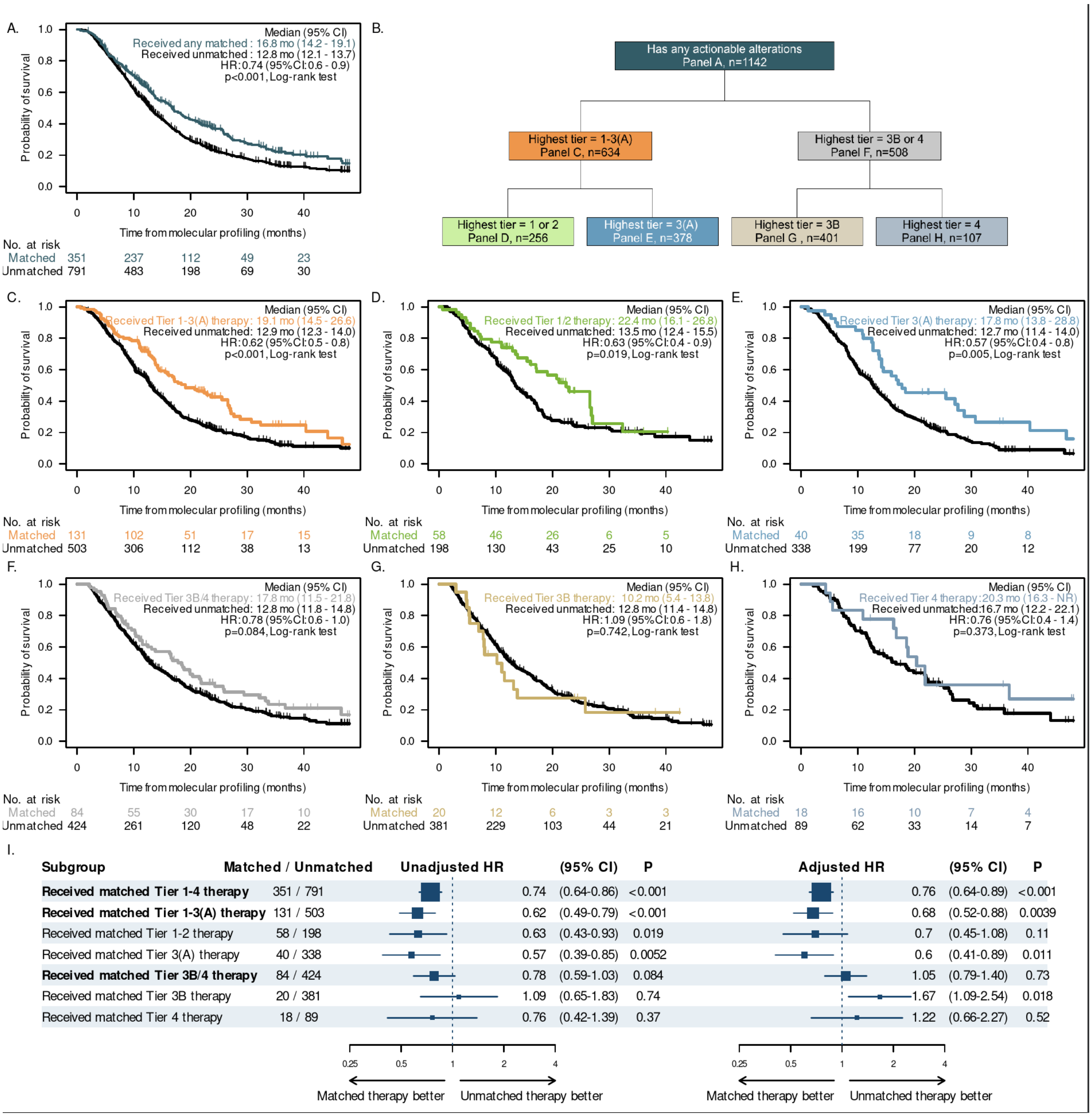

eFigure 10. Tier-matched sensitivity analysis comparing survival for genomically matched versus unmatched therapies using TOPOGRAPH version 20251018. Tier-matched analysis, using updated TOPOGRAPH knowledge base (version October 18, 2025) in Cohort B showing the difference in survival between patients within the same tier group who received genomically matched, versus unmatched, therapies. The results shows overall Panel (A) shows difference in OS between matched (Tier 1-4) versus unmatched therapy with unadjusted HR, and Panel (B) illustrates the stratification of groups by actionability in these tier-match analyses. Patients with highest genomically matched TOPOGRAPH tier in the (C) clinically active tier group (Tiers 1-3), (D) approved therapies (Tiers 1-2), (E) unapproved therapies with positive data shown in prospective trial (Tier 3), (F) investigational therapy tier group (Tier 3B and 4), (G) biomarker-matched therapy in non-cognate

© 2026 Lin FP et al. JAMA Oncol.

cancer type (Tier 3B), and (H) therapies with only preclinical or early clinical evidence (Tier 4). (I) Forest plot showing adjusted HR considering time-to-matched therapy and time-to-unmatched therapy in regression models. Hazard ratios were adjusted for the time-to-initiation of the most active subsequent therapies, age, ECOG performance status at the time of consent, cancer type, and whether the patient had previously received a therapy that matched the corresponding TOPOGRAPH tier or tier group.

© 2026 Lin FP et al. JAMA Oncol.

eFigure 11. Landmark analyses of survival between matched, unmatched, and untreated groups after genomic profiling.

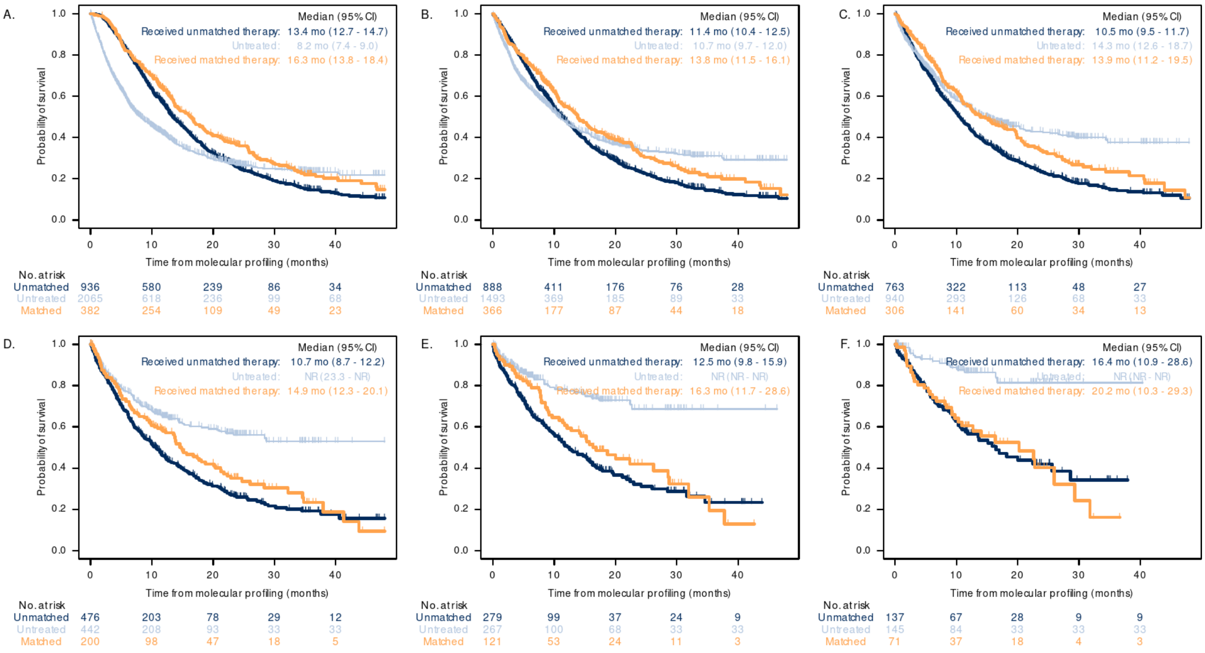

G.

Landmark time	Cohort A (Untreated)				Cohort B (Matched)			
	HR(Death)			P	HR (death)			P
	Estimate	lower .95	upper .95		Estimate	lower .95	upper .95	
Unrestricted	1.33	1.21	1.46	<0.001	0.82	0.71	0.95	0.009
3 months	0.93	0.84	1.04	0.203	0.82	0.71	0.96	0.011
6 months	0.71	0.62	0.81	<0.001	0.76	0.64	0.90	0.002
12 months	0.51	0.42	0.62	<0.001	0.80	0.64	1.00	0.051
18 months	0.37	0.27	0.50	<0.001	0.78	0.58	1.07	0.121
24 months	0.25	0.15	0.44	<0.001	1.02	0.67	1.55	0.933

Landmark survival analyses of three groups of patients (Cohort B, matched; Cohort B, unmatched; Cohort A, untreated) were conducted at 0, 3, 6, 12, 18, and 24 months (Panels A-F). The Schoenfeld residual analysis showed that proportional hazards assumption was not met at 0, 3, 6, 9, 12, and 18 months, indicating that the hazard ratios varied over these time intervals between the three groups. However, the assumption became valid after 24 months, suggesting that the hazard ratios stabilized and remained constant from this point onward (p=0.5, chi-square-test). This analysis highlights the significant difference in patient characteristics between the Cohorts. In Cohort A, the reason behind why a patient did not undergo further therapy were not feasible to be collected. However, it would consist of a mixture of patients with mainly patients with truly poor prognosis and patients with indolent cancers without therapy referred for sequencing, whom systemic therapy is not immediately required after molecular profiling. The demographics and survival characteristics between Cohorts A and B have highlighted that most of the early differences in outcome between the

© 2026 Lin FP et al. JAMA Oncol.

two cohorts were driven by the selection of poorer-risk patients for no further therapy (Cohort A, Panel G, showing hazard ratio for death versus the unmatched therapy). In contrast, cohort B included patients who required and were able to receive further systemic therapy and were a more homogeneous group, with compatible magnitude of HR to the regression analyses that incorporated the time-dependent covariate.

© 2026 Lin FP et al. JAMA Oncol.

eTable 1. Characteristics of the solid tumour cohort in the MoST study

Characteristic	Value	Cohort A (n=2065)		Cohort B (n=1318)		Total (N=3383)		P*
		N	(%)	N	(%)	N	(%)	
Age group (years)	<20	8	(0.4)	10	(0.8)	18	(0.5)	0.0014
	20-34	148	(7)	119	(9)	267	(7.9)	
	35-44	231	(11)	171	(13)	402	(12)	
	45-54	383	(19)	262	(20)	645	(19)	
	55-64	570	(28)	366	(28)	936	(28)	
	65-74	529	(26)	310	(24)	839	(25)	
	75-84	186	(9)	79	(6)	265	(7.8)	
	>=85	10	(0.5)	1	(0.1)	11	(0.3)	
Sex	Female	1072	(52)	720	(55)	1792	(53)	0.13
	Male	993	(48)	598	(45)	1591	(47)	
ECOG group	ECOG 0	884	(43)	706	(54)	1590	(47)	<0.001
	ECOG 1	1072	(52)	575	(44)	1647	(49)	
	ECOG 2	94	(5)	31	(2)	125	(3.7)	
	ECOG 3	1	(<0.1)	1	(0.1)	2	(0.1)	
	Not reported	1	(<0.1)	0	(0)	1	(0)	
Rare class	Common	442	(21)	285	(22)	727	(21)	<0.001
	Less Common	167	(8)	62	(5)	229	(6.8)	
	Rare	1456	(71)	971	(74)	2427	(72)	
Sequencing panel	TSO500	924	(45)	295	(22)	1219	(36)	<0.001
	TST170	568	(28)	535	(41)	1103	(33)	
	FMI	427	(21)	328	(25)	755	(22)	
	Other	146	(7)	160	(12)	306	(9)	
Self-reported Ethnicity	Australian and New Zealander	886	(43)	643	(49)	1529	(45)	0.12
	Australian Aboriginal and Torres Strait Islander	33	(2)	19	(1)	52	(2)	
	British, Western and Northern European	619	(30)	322	(24)	941	(28)	
	Southern, Southeastern & Eastern European	162	(8)	111	(8)	273	(8)	
	Chinese Asian	101	(5)	61	(5)	162	(5)	
	Arab, North African and Middle Eastern	68	(3)	46	(3)	114	(3)	
	Southeast Asian	64	(3)	39	(3)	103	(3)	
	South and Central Asian	44	(2)	26	(2)	70	(2)	
	Central and South American	21	(1)	12	(0.9)	33	(1)	
	Southern and East African	19	(0.9)	10	(0.8)	29	(0.9)	
	Other North-East Asian	15	(0.7)	8	(0.6)	23	(0.7)	
	Pacific Islander and New Zealand Maori	11	(0.5)	11	(0.8)	22	(0.7)	
	North American	9	(0.4)	6	(0.5)	15	(0.4)	
	Not specified	13	(0.6)	4	(0.3)	17	(0.5)	
Cancer type	Sarcoma	354	(17)	300	(23)	654	(19)	<0.001
	Colorectal	205	(10)	141	(11)	346	(10)	
	Pancreas	185	(9)	103	(8)	288	(8.5)	
	High-grade gliomas	146	(7)	93	(7)	239	(7.1)	
	Breast	104	(5)	85	(6)	189	(5.6)	
	Ovarian	90	(4)	82	(6)	172	(5.1)	
	Biliary and gallbladder	113	(5)	57	(4)	170	(5)	
	Gastroesophageal	105	(5)	44	(3)	149	(4.4)	
	Unknown primary	79	(4)	54	(4)	133	(3.9)	
	Non-small cell lung	78	(4)	36	(3)	114	(3.4)	
	Endometrial	64	(3)	33	(3)	97	(2.9)	
	Prostate	53	(3)	40	(3)	93	(2.7)	
	Bladder	44	(2)	32	(2)	76	(2.2)	
	Head and neck	53	(3)	13	(1.0)	66	(2)	
	Small bowel including appendiceal	42	(2)	20	(2)	62	(1.8)	
	Salivary gland	40	(2)	21	(2)	61	(1.8)	
	Renal	31	(2)	15	(1)	46	(1.4)	
	Other central nervous system	28	(1)	16	(1)	44	(1.3)	
	Thyroid	32	(2)	12	(0.9)	44	(1.3)	
	Endocrine gland	19	(0.9)	19	(1)	38	(1.1)	
	Cervical	25	(1)	12	(0.9)	37	(1.1)	

© 2026 Lin FP et al. JAMA Oncol.

Characteristic	Value	Cohort A (n=2065)		Cohort B (n=1318)		Total (N=3383)		P*
		N	(%)	N	(%)	N	(%)	
	Mesothelioma	21	(1)	10	(0.8)	31	(0.9)	
	Thymic epithelial	24	(1)	7	(0.5)	31	(0.9)	
	Gastrointestinal stromal	18	(0.9)	6	(0.5)	24	(0.7)	
	Anal	15	(0.7)	7	(0.5)	22	(0.7)	
	Hepatocellular	11	(0.5)	8	(0.6)	19	(0.6)	
	Small-cell lung	11	(0.5)	7	(0.5)	18	(0.5)	
	Neuroendocrine tumour	12	(0.6)	5	(0.4)	17	(0.5)	
	Germ cell tumour	8	(0.4)	6	(0.5)	14	(0.4)	
	Sex cord-gonadal stromal	8	(0.4)	6	(0.5)	14	(0.4)	
	Uveal melanoma	6	(0.3)	8	(0.6)	14	(0.4)	
	Non melanoma skin	8	(0.4)	5	(0.4)	13	(0.4)	
	Cutaneous melanoma	8	(0.4)	3	(0.2)	11	(0.3)	
	Vulval and vaginal	6	(0.3)	5	(0.4)	11	(0.3)	
	Peripheral nervous system	9	(0.4)	1	(0.1)	10	(0.3)	
	Non-melanoma skin	4	(0.2)	5	(0.4)	9	(0.3)	
	Penile and urethral	6	(0.3)	1	(0.1)	7	(0.2)	

Note: (*) Chi-square test for independence. No significant variation was observed among the three primary sequencing panels employed in this study coming to the proportion of patients receiving potentially actionable Tier 1-3(A) therapy: TSO500 (n=490, 40%), TST170 (n=398, 36%), and FoundationOne CDx (n=298, 39%, p=0.10, chi-squared test). No difference in self-reported ethnicity composition was observed.

© 2026 Lin FP et al. JAMA Oncol.

eTable 2. Highest actionable TOPOGRAPH tiers in patients with at least one potential therapy matched to the genomic profile.

TOPOGRAPH Tier	Cohort A Received no therapy after genomic profiling (n=1,808)		Cohort B Received any therapy after genomic profiling (N=1,195)		Total (N=3,003)	
	N	(%)	N	(%)	N	(%)
Clinically active tier group (N=1270)	744	(41)	526	(44)		
Tier 1	130	(6)	99	(8)	229	(8)
Tier 1B	54	(3)	49	(4)	103	(3)
Tier 2	144	(7)	88	(7)	232	(8)
Tier 3	416	(20)	290	(22)	706	(24)
Investigational tier group (N=1733)	1064	(59)	669	(56)		
Tier 3B	853	(41)	504	(38)	1357	(45)
Tier 4	211	(10)	165	(13)	376	(13)

Out of a total of 3,383 patients that had their genomic profiles analyzed, 3,003 (89%) had results that matched the TOPOGRAPH tier. Among these patients, 1,270 (37.5%) were found to have potentially actionable alterations based on the highest TOPOGRAPH tier that was matched to their genomic profile. There was no significant statistical difference observed between the tier groups in Cohorts A and B (p=0.13, Chi-square test, df=1).

© 2026 Lin FP et al. JAMA Oncol.

eTable 3. Median time-to-most active therapy in Cohort B.

Genomic actionability (Tier)	Tier-matched therapy received according to the actionability tier		Received an unmatched or a lower tier therapy	
	Delay (days)	IQR	Delay (days)	IQR
Untiered			79	36 – 169
Tiers 1 and 2	101	41 – 215	74.5	25 – 164
Tier 3(A)	51	28 – 144	82	33 – 152
Tier 3B	167 *	59 – 310	75	27 – 174
Tier 4	70.5	43 – 197	81	33 – 186.5

A Kruskal-Wallis test with 8 degrees of freedom revealed a statistically significant difference (p = 0.0311), suggesting possible differences in the time-to-most active therapy across genomic actionability tiers. Patients who received matched therapy were associated with significantly longer delays compared to those who received unmatched therapies in the subgroup of patients who had the highest actionable therapy of Tier 3B (p=0.011, asterisk, Dunn's test of rank sums corrected for multiple comparisons using the Benjamini-Hochberg method). IQR: interquartile range.

© 2026 Lin FP et al. JAMA Oncol.

eTable 4. Differences in gene coverage of various genomic profiling panels used in the MoST study.

Gene	Tier	Matched therapy received	N	Foundation One Cdx (N=735)			Illumina TST170 (N=1122)			Illumina TSO500 (N=1082)			Other panels (N=444)		
				D+	D-	(%)	D+	D-	(%)	D+	D-	(%)	D+	D-	(%)
ATRX	4	0	77	22	713	(3%)	3	1119	(0.3%)	40	1042	(3.7%)	14	430	(3.2%)
MTAP	4	0	73	71	664	(9.7%)	0	1122	(0%)	0	1082	(0%)	2	442	(0.5%)
KDM6A	4	0	40	11	724	(1.5%)	0	1122	(0%)	23	1059	(2.1%)	6	438	(1.4%)
NF2	4	1	34	16	719	(2.2%)	0	1122	(0%)	15	1067	(1.4%)	3	441	(0.7%)
RNF43	4	0	33	10	725	(1.4%)	0	1122	(0%)	18	1064	(1.7%)	6	438	(1.4%)
LRP1B	4	0	27	0	735	(0%)	1	1121	(0.1%)	19	1063	(1.8%)	9	435	(2%)
FRS2	4	0	27	1	734	(0.1%)	0	1122	(0%)	22	1060	(2%)	5	439	(1.1%)
MAP2K4	4	0	26	16	719	(2.2%)	0	1122	(0%)	8	1074	(0.7%)	2	442	(0.5%)
ARID2	4	0	20	0	735	(0%)	0	1122	(0%)	16	1066	(1.5%)	4	440	(0.9%)
NRG1	3	1	20	0	735	(0%)	9	1113	(0.8%)	6	1076	(0.6%)	5	439	(1.1%)
IRS2	4	0	16	8	727	(1.1%)	0	1122	(0%)	8	1074	(0.7%)	0	444	(0%)
KEAP1	4	0	15	5	730	(0.7%)	0	1122	(0%)	9	1073	(0.8%)	1	443	(0.2%)
FAT1	R2	0	15	0	735	(0%)	0	1122	(0%)	11	1071	(1%)	4	440	(0.9%)
ARID1B	4	0	14	0	735	(0%)	0	1122	(0%)	13	1069	(1.2%)	2	442	(0.5%)
GLI1	R2	0	14	0	735	(0%)	0	1122	(0%)	12	1070	(1.1%)	2	442	(0.5%)
FANCA	4	0	13	5	730	(0.7%)	0	1122	(0%)	5	1077	(0.5%)	3	441	(0.7%)
SLX4	4	2	13	0	735	(0%)	8	1114	(0.7%)	4	1078	(0.4%)	1	443	(0.2%)
HGF	3	0	11	10	725	(1.4%)	0	1122	(0%)	0	1082	(0%)	1	443	(0.2%)
ERCC2	4	0	11	0	735	(0%)	2	1120	(0.2%)	8	1074	(0.7%)	1	443	(0.2%)
SF3B1	4	0	11	5	730	(0.7%)	0	1122	(0%)	5	1077	(0.5%)	1	443	(0.2%)
FANCI	4	0	11	0	735	(0%)	8	1114	(0.7%)	3	1079	(0.3%)	1	443	(0.2%)
JAK1	R2	0	10	0	735	(0%)	0	1122	(0%)	9	1073	(0.8%)	1	443	(0.2%)
CD274	4	1	9	5	730	(0.7%)	0	1122	(0%)	3	1079	(0.3%)	2	442	(0.5%)
BRD4	4	0	9	7	728	(1%)	0	1122	(0%)	1	1081	(0.1%)	2	442	(0.5%)
PRKDC	4	0	9	0	735	(0%)	0	1122	(0%)	9	1073	(0.8%)	0	444	(0%)
LZTR1	4	0	9	0	735	(0%)	0	1122	(0%)	7	1075	(0.6%)	2	442	(0.5%)
FANCD2	4	2	9	0	735	(0%)	0	1122	(0%)	6	1076	(0.6%)	3	441	(0.7%)
MYB	4	0	8	3	732	(0.4%)	0	1122	(0%)	4	1078	(0.4%)	1	443	(0.2%)
MAPK1	R2	0	8	5	730	(0.7%)	0	1122	(0%)	1	1081	(0.1%)	2	442	(0.5%)
RAD51	4	0	8	0	735	(0%)	4	1118	(0.4%)	1	1081	(0.1%)	3	441	(0.7%)
RAD50	4	1	7	0	735	(0%)	0	1122	(0%)	3	1079	(0.3%)	4	440	(0.9%)
FLI1	4	0	7	0	735	(0%)	4	1118	(0.4%)	3	1079	(0.3%)	0	444	(0%)
KMT2C	4	0	7	0	735	(0%)	0	1122	(0%)	0	1082	(0%)	7	437	(1.6%)
DAXX	4	0	7	0	735	(0%)	0	1122	(0%)	2	1080	(0.2%)	5	439	(1.1%)
EPHA7	4	0	6	0	735	(0%)	0	1122	(0%)	6	1076	(0.6%)	0	444	(0%)
PDCD1LG2	4	0	6	4	731	(0.5%)	0	1122	(0%)	1	1081	(0.1%)	1	443	(0.2%)
ERRFI1	4	0	6	1	734	(0.1%)	0	1122	(0%)	4	1078	(0.4%)	1	443	(0.2%)
ERCC1	4	0	5	0	735	(0%)	3	1119	(0.3%)	2	1080	(0.2%)	0	444	(0%)
FAM175A	4	0	5	0	735	(0%)	2	1120	(0.2%)	3	1079	(0.3%)	0	444	(0%)
FANCG	4	0	5	3	732	(0.4%)	0	1122	(0%)	1	1081	(0.1%)	1	443	(0.2%)
RASA1	4	0	5	0	735	(0%)	0	1122	(0%)	5	1077	(0.5%)	0	444	(0%)
EIF1AX	4	0	5	0	735	(0%)	0	1122	(0%)	5	1077	(0.5%)	0	444	(0%)
YAP1	4	0	5	0	735	(0%)	0	1122	(0%)	5	1077	(0.5%)	0	444	(0%)
SDHB	4	0	4	0	735	(0%)	0	1122	(0%)	1	1081	(0.1%)	3	441	(0.7%)
SUFU	R2	0	4	1	734	(0.1%)	0	1122	(0%)	2	1080	(0.2%)	1	443	(0.2%)
PPM1D	4	0	4	0	735	(0%)	0	1122	(0%)	3	1079	(0.3%)	1	443	(0.2%)
FANCC	4	0	4	3	732	(0.4%)	0	1122	(0%)	0	1082	(0%)	1	443	(0.2%)
RIT1	R2	0	4	0	735	(0%)	0	1122	(0%)	4	1078	(0.4%)	0	444	(0%)
BCL6	4	0	4	0	735	(0%)	4	1118	(0.4%)	0	1082	(0%)	0	444	(0%)
SUZ12	4	0	4	0	735	(0%)	0	1122	(0%)	4	1078	(0.4%)	0	444	(0%)
H3F3A	4	0	4	1	734	(0.1%)	0	1122	(0%)	3	1079	(0.3%)	1	443	(0.2%)

© 2026 Lin FP et al. JAMA Oncol.

Gene	Tier	Matched therapy received	N	Foundation One Cdx (N=735)			Illumina TST170 (N=1122)			Illumina TSO500 (N=1082)			Other panels (N=444)		
				D+	D-	(%)	D+	D-	(%)	D+	D-	(%)	D+	D-	(%)
BCL2L11	R2	0	4	0	735	(0%)	0	1122	(0%)	3	1079	(0.3%)	2	442	(0.5%)
POLD1	4	0	4	0	735	(0%)	0	1122	(0%)	3	1079	(0.3%)	1	443	(0.2%)
ARAF	4	0	3	2	733	(0.3%)	0	1122	(0%)	0	1082	(0%)	1	443	(0.2%)
XPO1	R2	0	2	0	735	(0%)	1	1121	(0.1%)	1	1081	(0.1%)	0	444	(0%)
RAC1	4	0	2	1	734	(0.1%)	0	1122	(0%)	0	1082	(0%)	1	443	(0.2%)
FH	4	0	2	0	735	(0%)	0	1122	(0%)	2	1080	(0.2%)	1	443	(0.2%)
CD276	4	0	2	0	735	(0%)	0	1122	(0%)	2	1080	(0.2%)	0	444	(0%)
MAPK3	R2	0	1	0	735	(0%)	0	1122	(0%)	0	1082	(0%)	1	443	(0.2%)

The number of patients who received matched therapy based on the molecular profiling results is shown in the table. Eight patients were treated on the basis of the investigational biomarker. The shaded area indicates that gene targets are not present in the corresponding panel. Abbreviations: D+: gene alteration detected; D-: gene alteration not detected. N: number of patients with alterations in 3,383 patients; Tier: highest TOPOGRAPH tier associated recorded in the knowledge base.

© 2026 Lin FP et al. JAMA Oncol.

eTable 5. Drug classes of matched and unmatched most active therapies in Cohort B

Genomically matched to clinically active tier group (Tiers 1-3, n=526)						Genomically matched to investigational tier group (Tier 3B / 4, n=669)					
Received a matched Tier 1-3(A) therapy (n=116)			Received only unmatched therapy (n=410)			Received a matched Tier 3B or 4 therapy (n=133)			Received only unmatched therapy (n=536)		
**Drug classes**	**N**	**(%)**	**Drug classes**	**N**	**(%)**	**Drug classes**	**N**	**(%)**	**Drug classes**	**N**	**(%)**
PARP inh.	12	(10%)	Chemotherapy (ctx)	150	(37%)	PD-1/L1 mab. + PARP inh.	21	(16%)	Chemotherapy (ctx)	239	(44%)
EGFR mab. + ctx.	11	(9.5%)	CTLA-4 + PD-1/L1 mab.	31	(7.6%)	PD-1/L1 mab. + CDK4/6 inh.	19	(14%)	PD-1/L1 + CTLA-4 mab.	50	(9.3%)
PD-1/L1 mab.	11	(9.5%)	PD-1/L1 mab.	23	(5.6%)	CDK4/6 inh.	17	(13%)	PD-1/L1 mab.	40	(7.5%)
ERBB2 adc.	9	(7.8%)	ERBB2 adc.	19	(4.6%)	MTORC1 inh.	9	(6.8%)	KIT inh., ATP-competitive	18	(3.4%)
EGFR mab.	8	(6.9%)	VEGF mab. + ctx.	16	(3.9%)	PARP inh.	9	(6.8%)	VEGF mab.	17	(3.2%)
CDK4/6 inh.	7	(6.0%)	PD-1/L1 mab. + PARP inh.	14	(3.4%)	PD-1/L1 + CTLA-4 mab.	9	(6.8%)	IL-23 mab.	13	(2.4%)
RET inh.	6	(5.2%)	CTLA-4 mab.	11	(2.7%)	PD-1/L1 mab.	8	(6.0%)	PDGFR inh.	11	(2.1%)
pan-FGFR inh.	6	(5.2%)	VEGF mab.	11	(2.7%)	SMO inh.	6	(4.5%)	VEGF mab. + ctx.	11	(2.1%)
TRK inh.	5	(4.3%)	PARP inh.	9	(2.2%)	WEE1 inh.	3	(2.3%)	PD-1/L1 mab. + PARP inh.	9	(1.7%)
BRAF V600 inh. + EGFR mab.	4	(3.4%)	PD-1/L1 mab. + CDK4/6 inh.	8	(2.0%)	BRAF V600 inh. + MEK inh.	2	(1.5%)	VEGFR inh.	9	(1.7%)
KIT inh.	3	(2.6%)	CDK4/6 inh.	4	(1.0%)	RAF dimer inh. + MEK inh.	2	(1.5%)	SMO inh.	4	(0.7%)
SMO inh.	3	(2.6%)	ERBB2 mab. + ctx.	4	(1.0%)	KIF18A inh.	2	(1.5%)	VEGF inh.	4	(0.7%)
ERBB2 mab. + ctx.	3	(2.6%)	PD-1/L1 mab. + ctx.	4	(1.0%)	MEK inh.	2	(1.5%)	PD-1/L1 + TIGIT mab.	4	(0.7%)
PD-1/L1 mab. + TIGIT mab.	2	(1.7%)	CDK4/6 inh. + aromatase inh.	3	(0.7%)	MET inh., type 1	2	(1.5%)	PD-1/L1 mab. + ctx.	4	(0.7%)
BRAF V600 inh. + MEK inh.	2	(1.7%)	EGFR inh.	3	(0.7%)	RAF dimer inh.	2	(1.5%)	aromatase inh.	4	(0.7%)
BRAF V600 inh. + MEK inh. + EGFR mab.	2	(1.7%)	RAF dimer inh. + MEK inh.	3	(0.7%)	PD-1/L1 mab. + ctx.	2	(1.5%)	bispecific PD-1/CTLA-4 ab.	4	(0.7%)
EGFR inh.	2	(1.7%)	MEK inh.	3	(0.7%)	Platinum-based ctx.	2	(1.5%)	BCL2 inh.	3	(0.6%)
FGFR1/2/3 inh.	2	(1.7%)	MTORC1 inh.	3	(0.7%)	pan-FGFR inh.	2	(1.5%)	CDK4/6 inh.	3	(0.6%)
MET inh., type 1	2	(1.7%)	SMO inh.	3	(0.7%)	PD-1/L1 mab. + 177Lu-PSMA-rcj.	1	(0.8%)	MEK inh.	3	(0.6%)
BRAF V600 inh.	1	(0.9%)	VEGFR inh.	3	(0.7%)	CDK4/6 inh. + ERK inh.	1	(0.8%)	MTORC1 inh.	3	(0.6%)
BRAF V600 inh. + EGFR mab. + ctx.	1	(0.9%)	ERBB2 mab.	3	(0.7%)	CDK4/6 inh. + aromatase inh.	1	(0.8%)	VEGF inh. + PD-1/L1 mab.	3	(0.6%)
PD-1/L1 mab. + IL-2 variant	1	(0.9%)	soluble CD80 fusion protein	3	(0.7%)	EGFR inh.	1	(0.8%)	bispecific PD-1/VEGF ab.	3	(0.6%)
KIT inh.	1	(0.9%)	177Lu-PSMA-rcj.	2	(0.5%)	ERK inh.	1	(0.8%)	bispecific PD-L1/CTLA-4 ab.	3	(0.6%)
KRAS G12C inh. + SHP2 inh.	1	(0.9%)	ALK inh.	2	(0.5%)	EZH2 inh.	1	(0.8%)	AXL inh.	2	(0.4%)
MTORC1 inh.	1	(0.9%)	B7-H3 adc.	2	(0.5%)	MEK inh. + SMO inh.	1	(0.8%)	Multikinase inh.	2	(0.4%)
PARP inh. + PD-1/L1 mab.	1	(0.9%)	EGFR mab.	2	(0.5%)	PARP inh. + pan-AKT inh.	1	(0.8%)	KIT inh.	2	(0.4%)
PARP inh. + ctx.	1	(0.9%)	ERBB2 mab. + ctx.	2	(0.5%)	PKC inh.	1	(0.8%)	PARP inh.	2	(0.4%)
PDGFRA inh.	1	(0.9%)	PD-1/L1 mab. + angiogenesis inh.	2	(0.5%)	TEAD inh.	1	(0.8%)	PDGFRA inh.	2	(0.4%)
ROS1 inh.	1	(0.9%)	PD-1/L1 mab. + SHP2 inh.	2	(0.5%)	EGFR adc. + VEGF mab. + ctx.	1	(0.8%)	TGF-beta receptor inh.	2	(0.4%)
WEE1 inh.	1	(0.9%)	PD-1/L1 mab. + VEGF inh.	2	(0.5%)	EGFR adc. + ctx.	1	(0.8%)	A2aR and A2bR inh. + ctx.	2	(0.4%)
ERBB2 mab. doublet	1	(0.9%)	aromatase inh.	2	(0.5%)	IL15 mab. + PD-1/L1 mab.	1	(0.8%)	EGFR adc.	2	(0.4%)
ERBB3 mab.	1	(0.9%)	CDK4/6 inh. + SERD	2	(0.5%)	OX40 mab. + PD-1/L1 mab.	1	(0.8%)	antiandrogen, nonsteroidal	2	(0.4%)
PD-1/L1 mab. + TIM3 mab.	1	(0.9%)	CRAF inh. + KIT inh.	2	(0.5%)				progestogen analogue	2	(0.4%)
PD-1/L1 mab. + ctx.	1	(0.9%)	ERK inh.	2	(0.5%)				SERD	2	(0.4%)
bispecific c-Met/EGFR ab.	1	(0.9%)	RAF dimer inh.	2	(0.5%)				steroidogenesis inh.	2	(0.4%)
			Others	53	(13%)				Others	50	(9.3%)

For the unmatched therapy groups, treatment regimens with a frequency of less than two were included in the Others category. Abbreviations: ab.: antibody; adc: antibody-drug conjugate; ctx: chemotherapy; GnRH: Gonadotropin-releasing hormone; mab.: monoclonal antibody; inh. Inhibitor; rcj.: radioconjugate; A2aR/A2bR: adenosine A2A/2B receptors; AXL: receptor tyrosine kinase *Axl*; B7-H3: B7 homolog 3 protein (B7-H3, CD276); BCL2:

© 2026 Lin FP et al. JAMA Oncol.

B-cell lymphoma 2 gene; BRAF: proto-oncogene *B-Raf*; CDK: Cyclin-dependent kinase; CRAF: proto-oncogene c-RAF; CTLA-4: cytotoxic T-lymphocyte associated protein 4; EGFR: Epidermal growth factor receptor; ERBB2: Receptor tyrosine-protein kinase *erbB-2*; ERBB3: Receptor tyrosine-protein kinase *erbB-3*; ERK: mitogen-activated protein kinase 1; EZH2: enhancer of zeste homolog 2; FGFR: fibroblast growth factor receptor; IL: interleukin; KIF18A: kinesin family member 18A; KIT: Proto-oncogene *c-KIT*; MEK: mitogen-activated protein kinase kinase; MET: tyrosine-protein kinase *Met*; MTORC1: mammalian target of rapamycin complex 1; OX40: Tumor necrosis factor receptor superfamily, member 4; PARP: Poly (ADP-ribose) polymerase; PD-1/L1; programmed death protein 1/PD-ligand 1; PDGFRA: platelet-derived growth factor receptor A; PKC: Protein kinase C; PSMA: prostate-specific membrane antigen; RAF: RAF kinases; RET: proto-oncogene RET; ROS1: ROS proto-oncogene 1; SERD: selective estrogen receptor degrader; SHP2: Src homology region 2; SMO: Smoothened; TEAD: Transcriptional enhancer factor TEF-1; TGF: Transforming growth factor; TIGIT: T cell immunoreceptor with Ig and ITIM domains; TIM3: hepatitis A virus cellular receptor 2; TRK: Tropomyosin receptor kinase; VEGF: Vascular endothelial growth factors; WEE1: checkpoint kinase Wee1.

© 2026 Lin FP et al. JAMA Oncol.
