# Supplementary material for: Genomic Therapy Matching in Rare and Refractory Cancers
Source: JAMA Oncol. 2026 Mar 5;12(5):458–67. doi: 10.1001/jamaoncol.2026.0127 (PMC12964253; doi:10.1001/jamaoncol.2026.0127)
Supplement: Supplement 2. — Nonauthor Collaborators. The Australian Molecular Screening and Therapeutic program investigators and contributors [file jamaoncol-e260127-s002.pdf]

Supplemental Online Content: Nonauthor Collaborators
*First name, last name, and suffix (if applicable) are required and will appear in PubMed.

*Group Name(s): Australian Molecular Screening and Therapeutic program Investigators and Contributors							
***First Name and Middle Initial(s)**	***Last Name**	***Suffix (eg, Jr, III)**	Academic Degrees	Institution	Location (city, state/province, country)	Role or Contribution, eg, chair, principal investigator	Group (if more than 1 Group listed in the byline) and/or Subgroup (eg, Steering Committee)
Brett G	Hughes			The Prince Charles Hospital	Brisbane, QLD, Australia	Principal investigator	
Malinda	Itchins			Royal North Shore Hospital	Sydney, NSW, Australia	Principal investigator	
Chris	Karapetis			Flinders Medical Centre	Adelaide, SA, Australia	Principal investigator	
Steven	Lane			QIMR Berghofer and Royal Brisbane and Women's Hospital	Brisbane, QLD, Australia	Principal investigator	
Geoffrey	Peters			Canberra Hospital	Canberra, ACT, Australia	Principal investigator	
Sagun	Parakh			Austin Hospital	Melbourne, VIC, Australia	Principal investigator	
David	Ross			Royal Adelaide Hospital	Adelaide, SA, Australia	Principal investigator	
Katrin	Sjoquist			St George Hospital	Sydney, NSW, Australia	Principal investigator	
Mark	Shackleton			The Alfred Hospital	Melbourne, VIC, Australia	Principal investigator	
Adnan M	Nagrial			Westmead Hospital	Sydney, NSW, Australia	Principal investigator	
Melissa	Moore			St Vincent's Hospital	Melbourne, VIC, Australia	Principal investigator	
Maggie	Moore			The Alfred Hospital	Melbourne, VIC, Australia	Principal investigator	
Craig	Underhill			Border Medical Oncology	Wodonga, VIC, Australia	Principal investigator	
Hayley	Barker			Garvan Institute of Medical Research	Sydney, NSW, Australia	Contributor	
Emily	Collignon			Garvan Institute of Medical Research	Sydney, NSW, Australia	Contributor	
Laura	Conole			Garvan Institute of Medical Research	Sydney, NSW, Australia	Contributor	
Mark	Cowley			Garvan Institute of Medical Research	Sydney, NSW, Australia	Contributor	
Jenny	Gu			Garvan Institute of Medical Research	Sydney, NSW, Australia	Contributor	

Supplemental Online Content: Nonauthor Collaborators
*First name, last name, and suffix (if applicable) are required and will appear in PubMed.

*First Name and Middle Initial(s)	*Last Name	*Suffix (eg, Jr, III)	Academic Degrees	Institution	Location (city, state/province, country)	Role or Contribution, eg, chair, principal investigator	Group (if more than 1 Group listed in the byline) and/or Subgroup (eg, Steering Committee)
Elektra	Hajdu			Garvan Institute of Medical Research	Sydney, NSW, Australia	Contributor	
Erin	Heyer			Garvan Institute of Medical Research	Sydney, NSW, Australia	Contributor	
Luke	Hesson			Garvan Institute of Medical Research	Sydney, NSW, Australia	Contributor	
Tharindi	Ip			Garvan Institute of Medical Research	Sydney, NSW, Australia	Contributor	
Amelia	Mifsud			Garvan Institute of Medical Research	Sydney, NSW, Australia	Contributor	
Mark	Pinese			Garvan Institute of Medical Research	Sydney, NSW, Australia	Contributor	
Aaron	O'Grady			Garvan Institute of Medical Research	Sydney, NSW, Australia	Contributor	
Amy	Prawira			Garvan Institute of Medical Research	Sydney, NSW, Australia	Contributor	
Min R	Qiu			Garvan Institute of Medical Research	Sydney, NSW, Australia	Contributor	
Audrey	Silvestri			Garvan Institute of Medical Research	Sydney, NSW, Australia	Contributor	
Keith	Thornton			Garvan Institute of Medical Research	Sydney, NSW, Australia	Contributor	
Kelly	Walwyn			Garvan Institute of Medical Research	Sydney, NSW, Australia	Contributor	
Cheryll	Ye			Garvan Institute of Medical Research	Sydney, NSW, Australia	Contributor	
Anaiis	Zaratzian			Garvan Institute of Medical Research	Sydney, NSW, Australia	Contributor	
Nicola	Barrie			NHMRC Clinical Trials Centre, University of Sydney	Sydney, NSW, Australia	Contributor	

Supplemental Online Content: Nonauthor Collaborators
*First name, last name, and suffix (if applicable) are required and will appear in PubMed.

***First Name and Middle Initial(s)**	***Last Name**	***Suffix (eg, Jr, III)**	Academic Degrees	Institution	Location (city, state/province, country)	Role or Contribution, eg, chair, principal investigator	Group (if more than 1 Group listed in the byline) and/or Subgroup (eg, Steering Committee)
Michelle	Cummins			NHMRC Clinical Trials Centre, University of Sydney	Sydney, NSW, Australia	Contributor	
Yogita	Dheer			NHMRC Clinical Trials Centre, University of Sydney	Sydney, NSW, Australia	Contributor	
Kathleen	Harwood			NHMRC Clinical Trials Centre, University of Sydney	Sydney, NSW, Australia	Contributor	
Enam	Hoque			NHMRC Clinical Trials Centre, University of Sydney	Sydney, NSW, Australia	Contributor	
Sarah	Finlayson			NHMRC Clinical Trials Centre, University of Sydney	Sydney, NSW, Australia	Contributor	
Clarencia	Lie			NHMRC Clinical Trials Centre, University of Sydney	Sydney, NSW, Australia	Contributor	
Ian	Marschner			NHMRC Clinical Trials Centre, University of Sydney	Sydney, NSW, Australia	Contributor	
Rachael	Morton			NHMRC Clinical Trials Centre, University of Sydney	Sydney, NSW, Australia	Contributor	
James	Murray			NHMRC Clinical Trials Centre, University of Sydney	Sydney, NSW, Australia	Contributor	
Kelly	Nicholas			NHMRC Clinical Trials Centre, University of Sydney	Sydney, NSW, Australia	Contributor	
Tosin	Omotoso			NHMRC Clinical Trials Centre, University of Sydney	Sydney, NSW, Australia	Contributor	
Sachie	Pallimulla			NHMRC Clinical Trials Centre, University of Sydney	Sydney, NSW, Australia	Contributor	
Prapti	Pandya			NHMRC Clinical Trials Centre, University of Sydney	Sydney, NSW, Australia	Contributor	
Isabella	Richardson			NHMRC Clinical Trials Centre, University of Sydney	Sydney, NSW, Australia	Contributor	
Nick	Ristevski			NHMRC Clinical Trials Centre, University of Sydney	Sydney, NSW, Australia	Contributor	

Supplemental Online Content: Nonauthor Collaborators
*First name, last name, and suffix (if applicable) are required and will appear in PubMed.

***First Name and Middle Initial(s)**	***Last Name**	***Suffix (eg, Jr, III)**	Academic Degrees	Institution	Location (city, state/province, country)	Role or Contribution, eg, chair, principal investigator	Group (if more than 1 Group listed in the byline) and/or Subgroup (eg, Steering Committee)
Hayley	Thomas			NHMRC Clinical Trials Centre, University of Sydney	Sydney, NSW, Australia	Contributor	
Patrick	Wheeler			NHMRC Clinical Trials Centre, University of Sydney	Sydney, NSW, Australia	Contributor	
Vicki	Xie			NHMRC Clinical Trials Centre, University of Sydney	Sydney, NSW, Australia	Contributor	
Vera	Terry			Omico / Australian Genomic Cancer Medicine Centre	Sydney, NSW, Australia	Contributor	
Julia	Dobbins			Department of Genetics and Molecular Pathology and Centre for Cancer Biology, SA Pathology	Adelaide, SA, Australia	Investigator	
Anna L	Brown			Department of Genetics and Molecular Pathology, SA Pathology and Centre for Cancer Biology, University of South Australia, and Adelaide Medical School, University of Adelaide	Adelaide, SA, Australia	Investigator	
Alan	McGovern			Department of Genetics and Molecular Pathology, SA Pathology and Centre for Cancer Biology	Adelaide, SA, Australia	Contributor	
Rob	King			Centre for Cancer Biology	Adelaide, SA, Australia	Contributor	
Andreas W	Schreiber			Centre for Cancer Biology	Adelaide, SA, Australia	Contributor	
Rachael	Chang			Royal Adelaide Hospital	Adelaide, SA, Australia	Contributor	
Gonzalo	Tapia-Rico			Royal Adelaide Hospital	Adelaide, SA, Australia	Contributor	
Melinda	Whelan			Royal Adelaide Hospital	Adelaide, SA, Australia	Contributor	
Timothy	Humphries			School of Medicine, University of Western Australia	Perth, WA, Australia	Contributor	
