# Supplementary material for: Genomic Therapy Matching in Rare and Refractory Cancers
Source: JAMA Oncol. 2026 Mar 5;12(5):458–67. doi: 10.1001/jamaoncol.2026.0127 (PMC12964253; doi:10.1001/jamaoncol.2026.0127)
Supplement: Supplement 3. — Data Sharing Statement [file jamaoncol-e260127-s003.pdf]

Data Sharing Statement

Lin. Genomic Therapy Matching in Rare and Refractory Cancers. *JAMA Oncol*. Published March 05, 2026. doi:10.1001/jamaoncol.2026.0127

Data
Data available: Yes
Data types: Deidentified participant data
How to access data: Data availability: The primary dataset containing individual de-identified participant data is available via controlled access, subject to approval from the ethics committee and data custodians (University of Sydney and Australian Genomic Cancer Medicine Centre, Omico).
When available: With publication

Supporting Documents
Document types: Statistical/analytic code
How to access documents: Software and computer code: The software and database (TOPOGRAPH, https://topograph.info) used in this study are released as open source software on GitHub (https://github.com/fpylin/POTTR) for therapy tiering/classification, as described in the manuscript. The code is fully accessible to editors and reviewers.
When available: With publication

Additional Information
Who can access the data: Academic research.
Types of analyses: See above
Mechanisms of data availability: See above
